# Mutational cooperativity of *RUNX1::RUNX1T1 isoform 9a* and oncogenic *NRAS* in zebrafish myeloid leukaemia

**DOI:** 10.1242/bio.060523

**Published:** 2024-08-30

**Authors:** Robyn Lints, Christina A. Walker, Omid Delfi, Matthew Prouse, Mandy PohLui De Silva, Stefan K. Bohlander, Andrew C. Wood

**Affiliations:** ^1^ Leukaemia and Blood Cancer Research Unit, Department of Molecular Medicine and Pathology, University of Auckland, Auckland 1023, New Zealand; ^2^ Starship Child Health, Starship Blood and Cancer Centre, Auckland 1023, New Zealand

**Keywords:** NRAS(G12D), RUNX1::RUNXT1, Leukaemia, Model, Oncogene cooperation, Zebrafish

## Abstract

*RUNX1::RUNX1T1 (R::RT1)* acute myeloid leukaemia (AML) remains a clinical challenge, and further research is required to model and understand leukaemogenesis. Previous zebrafish *R::RT1* models were hampered by embryonic lethality and low penetrance of the malignant phenotype. Here, we overcome this by developing an adult zebrafish model in which the human *R::RT1* isoform *9a* is co-expressed with the frequently co-occurring oncogenic *NRAS^G12D^* mutation in haematopoietic stem and progenitor cells (HSPCs), using the *Runx1^+23^* enhancer. Approximately 50% of F0 *9a+NRAS^G12D^* transgenic zebrafish developed signs of haematological disease between 5 and 14 months, with 27% exhibiting AML-like pathology: myeloid precursor expansion, erythrocyte reduction, kidney marrow hypercellularity and the presence of blasts. Moreover, only *9a+NRAS^G12D^* transplant recipients developed leukaemia with high rates of mortality within 40 days, inferring the presence of leukaemia stem cells. These leukaemic features were rare or not observed in animals expressing either the *NRAS* or *9a* oncogenes alone, suggesting 9a and NRAS cooperation drives leukaemogenesis. This novel adult AML zebrafish model provides a powerful new tool for investigating the basis of R::RT1 - NRAS cooperativity with the potential to uncover new therapeutic targets.

## Teaser

**Summary:** We developed a novel model of acute myeloid leukaemia in adult zebrafish where *RUNX1:RUNX1T1* and *NRAS(G12*D) cooperate to increase penetrance and self-renewal to create a leukaemia that can be transplanted.

## INTRODUCTION

Acute myeloid leukaemias (AML) are an aggressive and heterogeneous group of cancers characterised by the accumulation of immature myeloid cells, and impairment of normal haematopoiesis. In WHO diagnostic criteria, translocation t(8;21) (q22;q22) is an AML defining genetic abnormality (Khoury et al., 2022), accounting for 11‒32% of paediatric and 4‒10% of adult AMLs (Bolouri et al., 2018; Chang et al., 2000; Grimwade et al., 1998; Ustun et al., 2018). The core-binding factor subunit gene *RUNX1* (aka *AML1*, *CBFA2*), a transcription factor essential for haematopoietic differentiation and myeloid maturation, is translocated from chromosome 21q22.12 to the transcriptional corepressor *RUNX1T1* locus (aka *ETO*, *CBFA2T1*) on 8q21 (Bruford et al., 2021; Lutterbach et al., 1998; Okuda et al., 1996). The resultant *RUNX1::RUNX1T1* (*R::RT1*) oncofusion gene encodes the DNA-binding domain of *RUNX1* fused in-frame to *RUNX1T1*, thereby converting the RUNX1 transcriptional activator into a transcriptional repressor with dominant negative activity at RUNX1 DNA-binding sites (Gelmetti et al., 1998; Wang et al., 1998). The oncofusion protein impairs differentiation and apoptosis and may predispose to additional genetic and epigenetic alterations that lead to leukaemia (Al-Harbi et al., 2020). Although t(8;21) is considered a comparatively favourable prognostic factor, relapse is frequent and in a recent international cohort of ∼250 adult *R::RT1* AMLs, median overall survival was only 31 months (Borthakur and Kantarjian, 2021; Ustun et al., 2018). In addition, treatment of AML can cause life-shortening and life-limiting side effects in survivors, including cardiotoxicity and secondary malignant neoplasia (Abrahão et al., 2021; Feijen et al., 2019; Turcotte et al., 2019). Thus, further research is required to understand the molecular pathogenesis of *R::RT1* AMLs to improve outcomes (Al-Harbi et al., 2020).

Early efforts to develop murine R::RT1 AML models revealed that the knock-in *R::RT1/+* heterozygous state is embryonic lethal due to disruption of foetal haematopoiesis (Okuda et al., 1998; Yergeau et al., 1997). To overcome this, alternative strategies were employed, such as inducible expression and murine bone marrow transplant (mBMT)-based methods. These typically expressed *R::RT1* or its splice variant *9a*, alone or with other gene mutations that frequently co-occur with t(8;21), such as *NRAS^G12D^* or *cKIT^N822K^* (Abdallah et al., 2021; Desai et al., 2020; Higuchi et al., 2002; Schessl et al., 2005; Yan et al., 2006; Yuan et al., 2001; Zhao et al., 2014; Zuber et al., 2009). The rationale to co-express mutations with *R::RT1* is that 95% of human t(8;21) AMLs harbour other mutations or chromosomal abnormalities. While co-occurring mutations may be passenger mutations that accumulate within the haematopoietic stem and progenitor population (HSPC) with age (Welch et al., 2012), recurring patterns of co-mutation and mutational exclusivity infer that cooperating oncogenic mutations act on distinct biological pathways to drive AML leukaemogenesis (Bolouri et al., 2018; Christen et al., 2019; Duployez et al., 2016; Faber et al., 2016; Ley et al., 2013; Papaemmanuil et al., 2016; Patel et al., 2012). In children diagnosed with t(8;21) AMLs at 3 to 12 years of age, t(8;21) was detectable in newborn blood samples, consistent with the acquisition of additional mutations over a latency period (Wiemels et al., 2002). In addition to somatically acquired mutations, recent studies indicate that R::RT1 misappropriates developmentally-related factors to induce neoplastic transformation (Abdallah et al., 2021). Although animal models and clinical data demonstrate that *R::RT1* alone is necessary but nearly always insufficient for transformation, the molecular and cellular consequences of mutational cooperation are not fully elucidated or exploited clinically.

Zebrafish provide a genetically amenable system for studying haematological diseases due to conserved haematopoietic genetic networks, fecundity, and scalability (Gore et al., 2018; Jagannathan-Bogdan and Zon, 2013; Potts and Bowman, 2017). Broad expression of human *R::RT1* cDNA in embryonic zebrafish alters primitive haematopoiesis, biasing myeloid lineages towards granulocyte production with concomitant loss of erythrocyte fate, a trend also seen in patients and murine models (Yeh et al., 2008). However, the impact of *R::RT1* on zebrafish definitive haematopoiesis, mutational cooperation, and leukaemogenesis beyond the larval stage has yet to be fully explored. We hypothesised that restricting *R::RT1* expression to the putative cells of leukaemia origin, the HSPCs, would bypass early lethality and that its co-expression with a commonly co-occurring *NRAS^G12D^* mutation would lead to oncogenic cooperation and the establishment of AML in adult zebrafish. To this end, we developed transgenic zebrafish coexpressing cDNAs encoding human *R::RT1* splice variant *9a (9a)* and human mutant *NRAS^G12D^ (NRAS)* in HSPCs. We find that expression of *9a* with *NRAS*, but not expression of either oncogene alone, promotes a haematological phenotype consistent with AML. The marrow of AML *9a+NRAS* zebrafish is hypercellular and enriched in immature myeloid and blast-like cells but deficient in erythrocytes. By contrast, *9a*-only-expressing animals are healthy with no overt signs of abnormal haematopoiesis, while those expressing *NRAS^G12D^* alone show signs of blood disease that are associated with expansion of mature myeloid and lymphoid cells and a reduction in erythrocytes. Significantly, our zebrafish transplantation data infer that only *9a+NRAS* disease, and not *NRAS*-only disease, is transplantable and able to re-establish leukaemia in recipients. Taken together, our findings suggest that when co-expressed in the zebrafish marrow, *9a* and *NRAS* mutational cooperation significantly enhances the establishment of self-renewal, an essential leukaemia hallmark. This transgenic model provides a genetically amenable and scalable system for exploring t(8;21) AML and oncogenic cooperation.

## RESULTS

### 9a and NRAS differentially affect HSPC regulators in early development

**Fig. 1.**
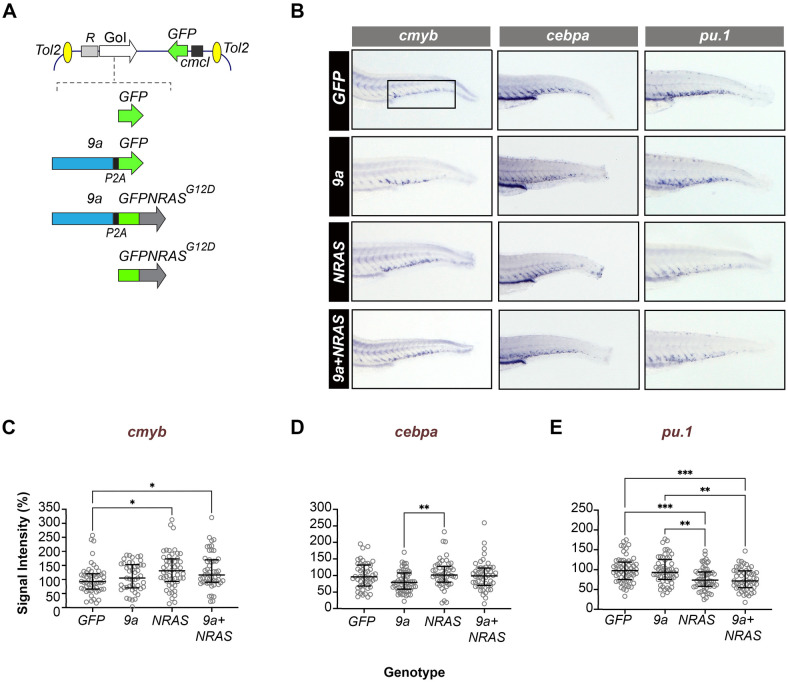
**HSPC-targeted expression of human oncogenes perturbs myeloid fate regulators in early definitive haematopoiesis.** (A) Schematic showing the structure of the Tol2 expression constructs used to generate F0 transgenic zebrafish. Top: generic organisation of the Tol2 destination vector showing the HSPC-specific *R1^+23^* expression driver (R), the gene of interest (GoI) and the heart-specific *cmcl2:GFP* transgenesis marker. Bottom: Schematic showing GoI details. *GFP* (control), cDNAs encoding human *RUNX1::RUNXT1 splice variant 9a (9a),* human *NRAS^G12D^* oncogene fused in frame to *GFP (GFPNRAS^G12D^)*. In *9a* and *9a+NRAS* constructs, the protein product of the second gene (GFP or GFPNRAS, respectively) is generated separately due to an intervening P2A “ribosome skipping” sequence (black box). (B) WISH of F0 mosaic animals at 72 hpf (hours post fertilisation) for *cmyb* (a definitive haematopoietic stem cell marker), *pu.1* and *cebpa* (myeloid program regulators; see Table S2 for probe details). Shown is staining in the CHT region of the larval tail (fetal liver equivalent; boxed) with probe (top) and genotype (left) indicated. (C–E) Quantitative analysis of *in situ* probe signal intensity in the CHT (see Materials and Methods for details of the quantification process). Each circle corresponds to the normalized staining pixel intensity of a single animal for the probe indicated. The total number of animals shown per probe and genotype (∼50–60 animals) corresponds to two technical replicates. Statistical tests: Kruskal–Wallis (if non-Gaussian distribution) or ANOVA (if Gaussian distribution). *cmyb* probe: *, *P*=0.0114, 0.0399 (*GFP* versus *NRAS*; *GFP* versus *9a+NRAS,* respectively); *cepba* probe: **P=0.0055 (*9a* versus *NRAS*); *pu.1* probe: **, *P*<0.0031, 0.0013 (*9a* versus *NRAS, 9a* versus 9a+*NRAS*, respectively); ****, *P*<0.0007, 0.0003 (*GFP* versus *NRAS, GFP* versus 9a+*NRAS*, respectively).

The establishment of leukaemia involves competition between mutated and healthy HSPCs (Glait-Santar et al., 2015; Loberg et al., 2019), ultimately leading to multiple AML subclones (Chopra and Bohlander, 2019; Jan et al., 2012). To model mutational cooperation in leukaemogenesis, we explored the inherent cellular mosaicism of F0 transgenics to study biologically distinct animals (Blainey et al., 2014; Ung et al., 2015). In *R::RT1* AML patients, transcripts encoding full-length R::RT1 fusion protein and a C-terminal truncated variant, 9a, have been detected at varied ratios (Yan et al., 2006). There is no consensus regarding the prognostic significance of either isoform or their relative abundance (Agrawal et al., 2020; Jiao et al., 2009; Ommen et al., 2010). However, mouse modelling studies suggest that the 9a isoform may have greater oncogenic potency than full-length RUNX1::RUNXT1 (Yan et al., 2006). For this reason, we preferentially used the *9a* variant in our expression constructs. We generated four transgenes: *9a-P2A-GFPNRAS^G12D^ (9a+NRAS)*, *9a-P2A-GFP (9a), GFPNRAS^G12D^ (NRAS)*, and *GFP* (Fig. 1A; Fig. S1; Table S1; see also, the Materials and Methods section). To achieve HSPC-restricted expression, transgenes were placed under the control of the conserved *Runx1+23/24* enhancer element fused to the mouse *β-globin* minimal promoter (collectively abbreviated as *R1^+23^*) (Nottingham et al., 2007; Tamplin et al., 2015). Constructs were injected into one-cell-stage wild-type embryos, and resultant transgenic animals were either used for embryonic whole-mount RNA *in situ* hybridization studies (WISH) or allowed to continue developing where they were monitored daily for signs of disease for up to 16 months.

HSPC generation and development are highly conserved between zebrafish and humans (Jagannathan-Bogdan and Zon, 2013; Mahony and Bertrand, 2019). In zebrafish the HSPCs emerge from the haemogenic endothelium of the ventral dorsal aorta wall (aorta-gonad mesonephros equivalent) at ∼26 hpf, and travel to the caudal haematopoietic tissue (CHT, foetal liver equivalent) where they undergo expansion before migrating to seed the kidney marrow. *R1^+23^* -driven expression of GFP in HSPCs can be detected as early as 32 hpf, in the CHT (Tamplin et al., 2015). The CHT HSPCs are a heterogeneous population consisting of a mixture of stem-like and lineage-committed HSPC subgroups (Xia et al., 2021). Fully differentiated cells associated with the CHT (macrophages, monocytes and erythrocytes) derive from previous haematopoietic waves. To assess whether HSPC-targeted expression of *9a* and/or *NRAS* impacts CHT HSPC populations and differentiation, we examined expression of seven key haematopoietic genes at 72 hpf (3 dpf), using WISH (Fig. 1B–E; Fig. S2). These genes included stem cell marker, *cmyb*, and lineage restriction effectors that are known targets of R::RT1 in AML, such as *cepba* and *pu.1* (myeloid factors) and *gata1a* (erythroid) (see Table S2 for probe information) (Choi et al., 2006; Stengel et al., 2021). Signal intensity in the CHT was quantified by measuring pixel intensity while blinded to genotype (Fig. 1B–E; see Materials and Methods for details). Expression of stem cell marker *cmyb* was not modified by *9a* alone, but *cmyb* expression increased in *NRAS* and *9a+NRAS* embryos. Expression of myeloid marker *pu.1* was not modified by *9a* alone but was decreased in *NRAS* and *9a+NRAS*. The effects on *cepba* were less pronounced, with no significant differences in expression compared to *GFP* controls (Fig. 1B–E). Expression of *gata1a* and *scl* were not modified, but *NRAS* alone did lead to a reduction in expression of the globin encoding *hbbe1.1.* Expression of *lyz* was not modified (Fig. S2). Taken together, these results suggest that *9a* and *NRAS* have distinct effects on HSPC proliferation and lineage restriction with the most pronounced effect being observed with *NRAS* increasing expression of the stem cell marker *cmyb* and decreasing expression of a myeloid marker *pu.1*.

### HSPC-targeted expression of 9a with NRAS, or NRAS alone, decreases zebrafish adult survival

**Fig. 2.**
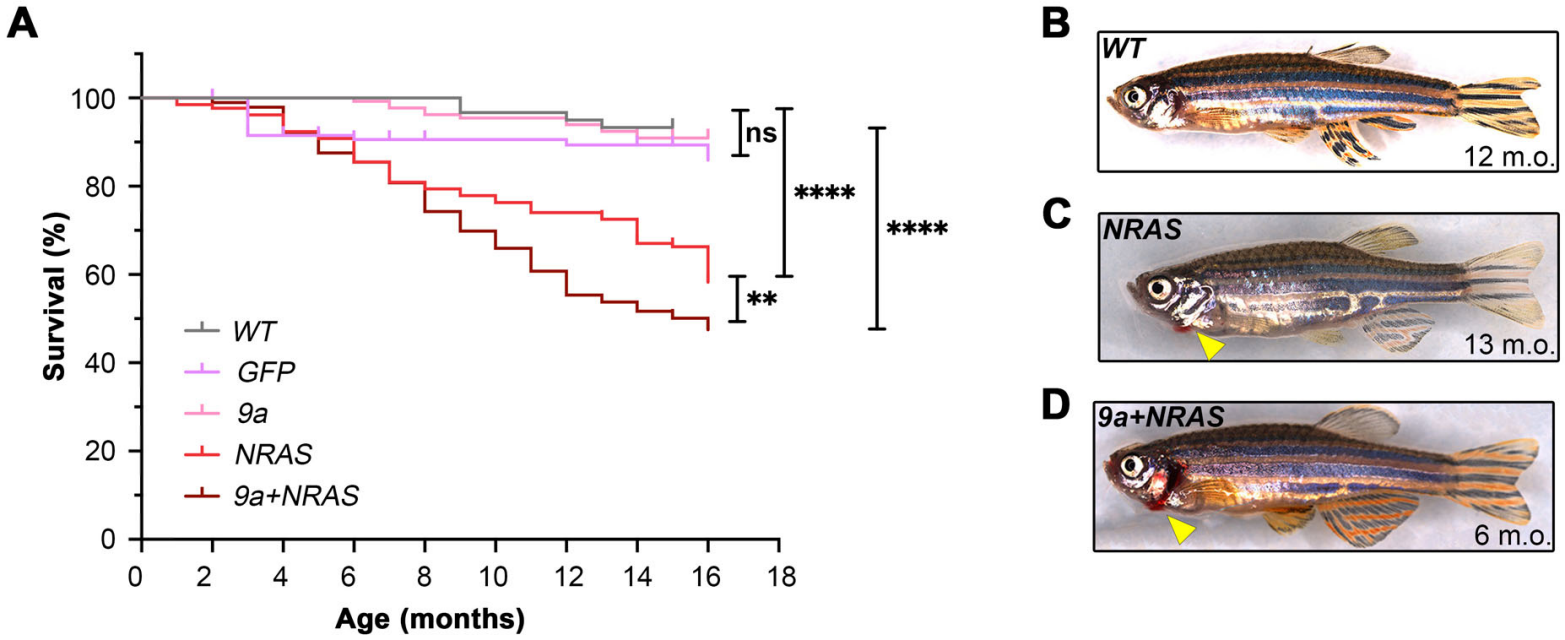
**HSPC-targeted expression of human oncogenic *9a* and *NRAS*, or *NRAS* alone, induces sickness and mortality in adult zebrafish.** (A) Kaplan–Meier Survival Curve of F0 transgenic populations generated by injection of the expression constructs shown in Fig. 1A. Data shown corresponds to analyses of 2 to 4 injected batches per genotype. Numbers of animals per genotype, n: Wild type *(WT)*, 60; *GFP*, 203; *9a*, 132; *NRAS*, 131; *9a+NRAS*, 384. Statistical test: Log-rank (Mantel-Cox) test: **P=0.009; ****P<0.0001; ns, not significant. (B–D) Whole animal images (stitched montages) of (B) healthy WT; (C) sick *NRAS* and (D) sick *9a+NRAS* F0 animals. Bleeding from gills (arrowhead) is seen only in sick oncogenic transgenics.

In adult transgenic populations we observed that survival of the *GFP* control cohort over a 16-month time frame was not significantly different from that of the wild-type background used to generate the F0 transgenic animals (*n*=13 deaths/203, 6%; Fig. 2A). Survival of the *9a*-only F0 cohort during the 16-month period was comparable to that of the *GFP* F0 population (*n*=12 deaths /132, 9%). Similarly, survival of F0 s expressing full-length *R::RT1* alone was also comparable to *GFP* controls (*n*=13 deaths/51, 25%; Fig. S3) suggesting that expression of this oncofusion in zebrafish does not overtly impact viability, regardless of isoform. By contrast, F0 animals expressing oncogenic *NRAS* alone showed significantly reduced survival (*n*=48 deaths/131, 37%; *P*=0.0024 *cf. GFP*) beginning at ∼3 months, displaying signs consistent with haematopoietic disease, that included reduced activity, bleeding from the gills, and rapid breathing (compare Fig. 2B–D). Although some features were potentially non-specific, oncogene expression was restricted to the HSPC compartment, and phenotypes are consistent with murine leukaemia models where oncogenic *NRAS* expression leads to symptomatic anaemia and thrombocytopaenia (Li et al., 2011; Wang et al., 2013, 2011). Like *NRAS* F0s, *9a+NRAS* F0 animals also showed significantly reduced survival from ∼3 months onwards, again associated with reduced activity, bleeding, and rapid breathing (*n*=202 deaths/385, 52%; *P*=0.0001 *cf. GFP*; Fig. 2D). Survival of F0 *9a+NRAS* fish was significantly lower than that of *NRAS* F0s (*P*=0.009 *cf. NRAS*) with 50% of *9a+NRAS* F0 succumbing by 16 months compared to 40% of *NRAS* animals. Therefore, *9a+NRAS* oncogene co-expression produced a more penetrant phenotype than expression of *NRAS* or *9a* alone. To investigate this, we further characterised the haematopoietic system of these animals.

### 9a+NRAS and NRAS transgenics exhibit distinct flow cytometric profiles

In adult zebrafish, the site of haematopoiesis is the kidney marrow, a tissue considered functionally analogous to the bone marrow of vertebrates (Jagannathan-Bogdan and Zon, 2013; Mahony and Bertrand, 2019). Like vertebrate bone marrow, the zebrafish kidney marrow harbours the HSPCs, their descendant precursor cells (immature, incompletely differentiated terminal blood cells), and mature blood cells yet to be released into the peripheral blood. To determine whether alterations in haematopoiesis were responsible for *9a+NRAS* or *NRAS* F0s sickness and mortality, we performed marrow cell flow cytometry, morphological analysis of marrow smears, RT-PCR for confirmation of transgene expression (Fig. S4), and histological analyses of whole animal tissue sections in GFP controls and oncogene transgenics.

**Fig. 3.**
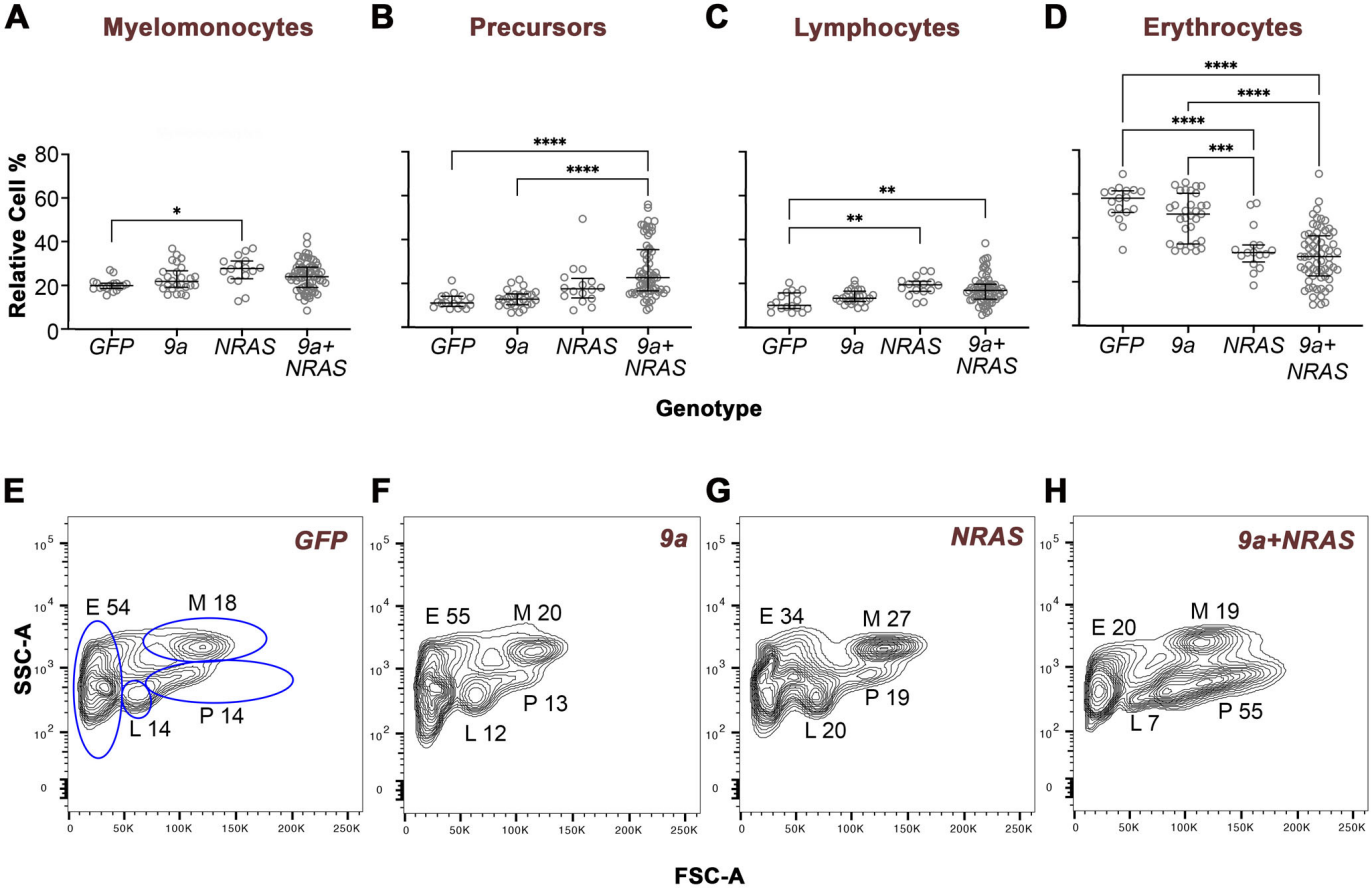
**Oncogenic F0 transgenics exhibit distinct WKM cell flow cytometry profiles.** Quantitative analyses of haematopoietic cell types in the zebrafish kidney marrow. WKM cells of the genotype indicated were subjected to forward versus side scatter (FSC/SSC) flow cytometry analysis, which resolves cells into four gated populations: myelomonocytes (M), progenitors/precursors (P), lymphocytes (L) and erythrocytes (E). For *NRAS* and *9a+NRAS* genotypes, animals were sick (age of sickness: 7–16 months); for *9a* or *GFP* animals were well and either age-matched with sick oncogenic animals or 16 months old. (A–D) Quantitative analyses of cell type percentages in the genotype indicated. For each animal, cell percentages were normalised so that the sum of all gates came to 100%. Each data point corresponds to a single animal. The total number of animals analysed per genotype, n: *GFP*, 17; *9a*, 24; *NRAS*, 16; *9a+NRAS*; 66. Statistical analysis: Ordinary one-way ANOVA, showing first (Q1), second (Q2, median) and third (Q3) quartiles. *, *P=*0.02/0.03; **, *P=*0.0015/0.0030; ***, *P=*0.0010; ****, *P<*0.0001. (E–H) Representative FSC/SSC flow cytometry profiles from individual animals of the genotype indicated. The fixed-gate dimensions (blue lines) shown in E were applied to all samples. The M, P, L and E numbers correspond to the relative percentage of cells in that gate. m.o., months old. See Fig. S7 for PCA of flow data.

Forward versus side scatter (FSC/SSC)-based cell flow cytometry resolves cells of the whole kidney marrow (WKM) into four major groups: progenitors/precursors (defined by the P gate), myelomonocytes (the M gate, which includes granulocytes, monocytes, basophils, and eosinophils), lymphocytes (L) and erythrocytes (E). The L gate harbours lymphocytes predominantly (Traver et al., 2003) (see Fig. S5A–C for cell morphology-flow gate correlation and Fig. S6 for *WT* versus *GFP* flow comparison). While *R1^+23^* GFP-marked HSPCs straddle the L and P gates, sorting of GFP-positive cells confirms that the majority of cells have a progenitor-like morphology (Fig. S5D) (Henninger et al., 2017; Tamplin et al., 2015)*.* In flow analysis, *9a* F0 marrows were not significantly different from that of age-matched *GFP* controls in the relative proportions of gated populations, consistent with the observation that *9a* F0 viability was not significantly impacted by oncofusion expression (Fig. 2A; Fig. 3A–D; compare Fig. 3E and Fig. 3F). In contrast, sick F0 *NRAS* marrow had a significantly higher fraction of myelomonocytes and lymphocytes but fewer erythrocytes compared to *9a* animals and *GFP* controls (Fig. S3A,C,D and flow profile example Fig. 3G), while the fraction of progenitor/precursors was not significantly different in these genotypes (Fig. 3B). This pattern (high M, high L and low E) was evident in 85% of F0 *NRAS* animals. An exception to this was a single *NRAS* F0 with a high proportion of progenitor/precursors (49%) and a low percentage of erythrocytes (18%) compared to *GFP* or *9a* genotypes. These phenotypes are reminiscent of the impact of *NRAS^G12D^* in murine systems where, depending on gene copy number, *NRAS^G12D^* promotes a form of myeloproliferative disease (MPD; characterised by lymphoproliferation with mature myeloid cell expansion and erythrocyte hypoplasia) or acute monocytic myeloid leukaemia (characterised by expansion of immature cells) (Li et al., 2011; Wang et al., 2011; Wang et al., 2015). In our zebrafish model, HSPC-targeted expression of oncogenic *NRAS* promotes a trend towards MPD in most F0s. Such disturbances in marrow haematopoiesis, including marked erythrocytopaenia, may contribute to reduced survival of *NRAS* F0s animals.

In contrast to *9a* and *NRAS* F0 animals, the *9a+NRAS* cohort produced more variable flow profiles. As seen among *NRAS* F0s, sick *9a+NRAS* animals had a significantly lower proportion of erythrocytes compared to *9a* and *GFP* controls, consistent with previous studies showing that R::RT1, like oncogenic NRAS, inhibits erythrocyte differentiation (Choi et al., 2006; Fenske et al., 2004; Schwieger et al., 2002; Tonks et al., 2003; Yeh et al., 2008). Lymphocytes were also significantly higher in *9a+NRAS* F0s compared to controls, possibly due to the action of NRAS (Fig. 3C). However, *9a+NRAS* and *NRAS* cohort flow profiles differed in two important respects. First, unlike *NRAS* F0s, myelomonocytes (M) were not significantly higher than *GFP* in *9a+NRAS* animals, consistent with the possibility that either 9a activity imposes a myeloid differentiation block or that NRAS expression levels are insufficient to drive myeloproliferation. Second, *9a+NRAS* F0s showed a significantly higher fraction of cells in the progenitors/precursors (P) gate compared to *NRAS* (*9a+NRAS* P gate median=22.7% *cf. NRAS* median=17.6%, *P*=0.02; *cf. 9a* median=12.7% and *GFP* median=11.1%, *P*=0.0001; Fig. 3A–D; Fig. 3H). Moreover, 27% of *9a+NRAS* animals (*n*=18/66 animals analysed) had a pronounced ‘AML-like’ flow profile, namely, progenitor/precursor percentages of 35% or higher that was invariably associated with low erythrocyte percentages (i.e. E<36%, that is, below the *NRAS* first quartile for E).

A principal component analysis (PCA) of flow data provides a visual representation of flow cytometry profiles in relation to genotypes (Fig. S7). The *9a+NRAS* cohort showed the greatest phenotypic heterogeneity, plotting as a continuum rather than a discrete cluster, with some animals aligning more with *9a* or *NRAS* alone genotypes, while those with AML-like profiles plotted more distantly from these. While our *NRAS* cohort demonstrates that *NRAS* alone can induce an AML-like phenotype, its frequency (1 in 16) is substantially lower than when *NRAS* is co-expressed with *9a* (1 in 4), even though *NRAS* is likely expressed at higher levels in *NRAS* animals than it is in *9a+NRAS* animals, due to the second gene position effects in the latter (Liu et al., 2017) (Fig. 1A).

### NRAS and 9a+NRAS expression alter marrow cell fate patterns

**Fig. 4.**
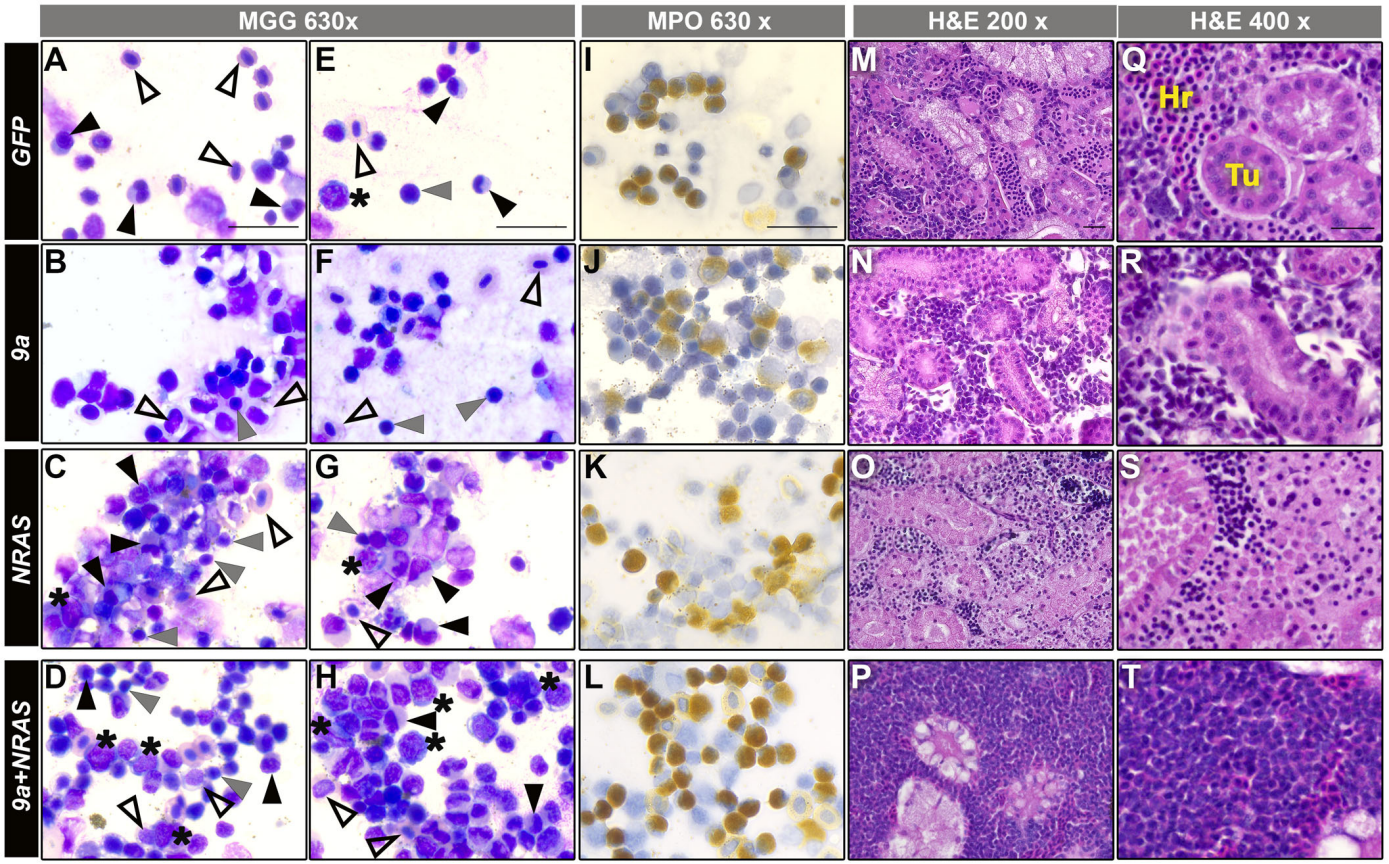
***9a+NRAS* F0 kidney marrow show blast cell expansion and hypercellularity.** (A–H) WKM cell smears of the genotypes indicated (side banners), stained with May Grünwald Giemsa (MGG) stain, magnification (top banner). Number of animals assessed per genotype, n: *GFP*, 22; *9a*, 31; *NRAS*, 11; *9a+NRA*S, 34. Blast cells (*); myelomonocytes (black triangles); lymphocytes (grey triangles); erythrocytes (open triangles). Scale bar: 20 μM. (I–L) WKM cell smears of the genotype indicated stained for myeloperoxidase (MPO) enzyme activity, a marker of myeloid granulocytic fate. Scale bar: 20 μM. See Fig. S8 for statistical analysis of genotypes. (M–T) Haematoxylin and Eosin (H&E) staining of kidney marrow tissue sections from animals of the genotype indicated. Hr, haematopoietic cells; Tu, kidney tubules. All sectioned *NRAS* and *9a+NRAS* animals were sick, while *9a* and *GFP* animals were well. Number of animals assessed per genotype, n: *GFP*, 2; *9a*, 5; *NRAS*, 4; *9a+NRAS*, 12. Sections reveal overt hypercellularity in 1 of 6 *9a+NRAS* animals (P, T, Fig. S9Aviii). Scale bars: 20 μM. For both smears and sections, *NRAS* and *9a+NRAS* animals shown were from sick, with age at time of sickness ranging from 7 to 16 months; *GFP* and *9a* were healthy and were either age-matched or 16 months old.

We stained marrow smears with May-Grünwald-Giemsa (MGG) to determine if oncogene expression impacted haematopoietic cell fates, as determined by morphological criteria (number of animals examined per genotype: *GFP*, 22; *9a*, 31; *NRAS*, 11; *9a+NRAS*, 34). Overall, the distribution of fates was highly consistent with flow cytometry patterns. The cellular composition of *9a* smears resembled age-matched *GFP* animals (Fig. 4B,F *cf.* A,E). Compared to *9a* and *GFP* animals, *NRAS* F0 smears harboured a higher proportion of mature myeloid cells (monocytes, eosinophils, and granulocytes), mature lymphocytes and immature erythroid cells (Fig. 4C,G). Significantly, a subset of *9a+NRAS* smears contained a high proportion of blast-like cells and immature myeloid cells, suggestive of AML disease (Fig. 4D,H). As noted above, R::RT1 AML is characterised by the accumulation of abnormal myeloid granulocytes exhibiting varied differentiation states (Arber et al., 2002; Jiang et al., 2020; Tonks et al., 2004; Westendorf et al., 1998). To determine if granulocytic cells were more abundant in *9a* or *9a+NRAS* F0s, we performed a cytochemical staining assay for myeloperoxidase (MPO) enzyme activity, a granulocytic fate marker (see Fig. 4I–L; Fig. S8). Although animals with the highest MPO percentages were associated with the *9a+NRAS* genotype, statistical analysis revealed no significant difference compared to other genotypes, possibly owing to the high degree of phenotypic heterogeneity among *9a+NRAS* animals.

### 9a+NRAS induces kidney marrow hypercellularity

To assess the impact of oncogene expression on the overall marrow architecture and cellular density, we fixed and sectioned animals of each genotype and stained tissue sections with Haematoxylin and Eosin (Fig. 4M–T; number of examined animals per genotype, n: *GFP*=2; *9a*=5; *NRAS=*4; *9a+NRAS*=12). The marrows of *9a, GFP* and sick *NRAS* animals were qualitatively similar for cell density (Fig. 4M–O,Q–S), suggesting that neither *NRAS-* nor *9a* expression alone induces overt cell proliferation. As observed in the flow analysis, *9a+NRAS* F0s displayed greater phenotypic variability. While some were comparable to *GFP*, *9a* and *NRAS*, 1 in 6 *9a+NRAS* F0 animals (i.e. *n*=2 of 12) showed significant marrow hypercellularity (Fig. 4P,T; Fig. S9A).

As an independent assessment of cell number, we resuspended the WKM cells from a non-overlapping set of animals (including those used in the transplant experiments described below) and counted total WKM cell number (Fig. S9B). While *NRAS* F0s WKM cell counts were higher than those of *9a* and *GFP*, *9a+NRAS* F0 counts were significantly greater (*NRAS* versus *GFP*, *P*=0.02; *9a+NRAS* versus *GFP*, *P*=0.0005). Furthermore, 1 in 8 (*n*=4/37) *9a+NRAS* F0s had counts exceeding the *NRAS* F0 maximum. This incidence of high *9a+NRAS* WKM cell counts was consistent with the incidence of *9a+NRAS* hypercellularity in sectioned animals (1 in 6) and AML-like flow profiles (1 in 4) in *9a+NRAS* F0s.

Taken together flow cytometry and histological data suggest that *NRAS* alone and *9a+NRAS* expression cause disturbances in marrow haematopoiesis that are sufficiently severe to account for the high rates of mortality in these cohorts. A major distinction between *NRAS-* and *9a+NRAS*-associated pathologies is the progenitor/precursor pool expansion and marrow hypercellularity in *9a+NRAS* animals. Overall, these data argue that when *NRAS* is co-expressed with *9a,* the probability of neoplastic transformation is increased.

### 9a+NRAS-, but not NRAS, cells can be transplanted and propagate the leukaemia-like phenotype

**Fig. 5.**
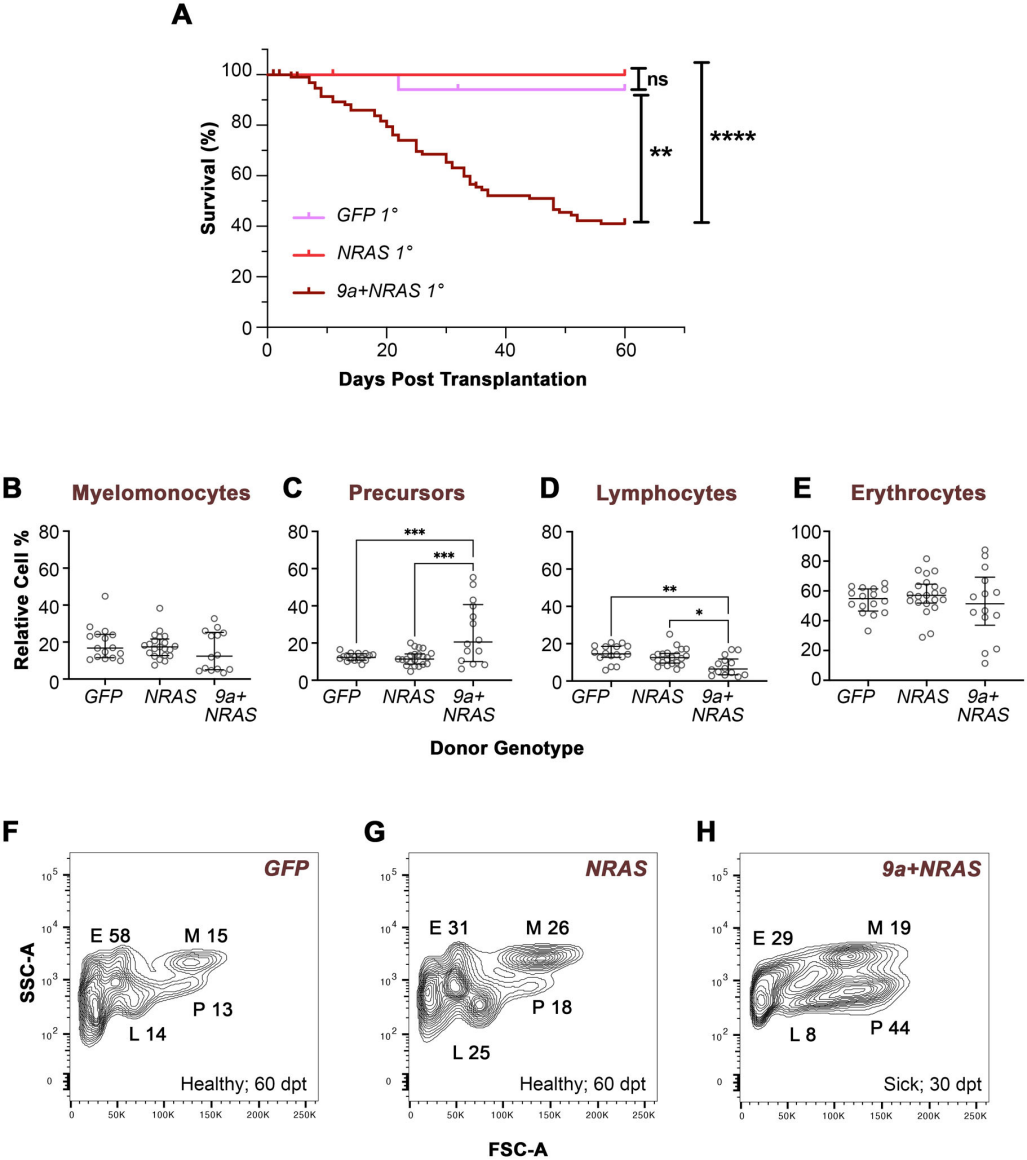
**Transplanted *9a+NRAS-*, but not *NRAS* disease, induces AML-like marrow cell profiles and sickness in recipient zebrafish.** (A) Kaplan–Meier survival plot of primary (1°) recipients transplanted with WKM cells from F0 donors of the genotype indicated. Donor age at the time of sickness ranged from 9 to 13 months. The number of donors (d) and 1° recipients (r) per genotype was as follows: *9a+NRAS* (d, 12; r, 72); *NRAS* (d, 5; r, 29); *GFP* (d, 4; r, 20). Statistical analysis: Log-Rank (Mantel-Cox) Test; **, *P<*0.0011; ****, *P<*0.0001; ns, not significant. (B–E) Quantitative analyses of WKM cells from primary recipient animals. Cells from recipients were subjected to FSC/SSC flow cytometry analysis, resolving cells into four gated populations. See Fig. 3 legend for details. Statistical analysis: Ordinary one-way ANOVA, showing first (Q1), second (Q2, median) and third (Q3) quartiles. *P=0.016; **P=0.004; ***P=0.0002/0.0006. (F–H) FSC/SSC flow cytometry plots from representative 1° recipients transplanted with cells the donor genotype indicated (see Fig. S10 for other examples). See Fig. S11 for RT-PCR confirmation of *NRAS* expression in recipient cells. dpt, days post-transplant.

While the decreased survival, hypercellularity, precursor/progenitor cells expansion and erythrocytopaenia in *9a+NRAS* animals represent evidence for an AML phenotype, a more definitive test is to demonstrate that the disease can be transplanted. Current models of cancer evolution posit that leukaemias harbour leukaemia stem cells (LSCs), which can establish leukaemia when transplanted into naïve healthy recipient animals (Chopra and Bohlander, 2019). To test whether either *9a+NRAS-* and *NRAS*-induced blood diseases might harbour LSCs, we performed transplant experiments using the standard allograft procedure employed in published zebrafish AML model studies (Fang et al., 2021; Le et al., 2007; Wang et al., 2023; Xu et al., 2020; Zhao et al., 2018). Specifically, we isolated WKM cells from sick *9a+NRAS* and *NRAS* donors and transplanted these into 5–10 wild-type recipient animals per donor (∼300,000 donor cells per recipient) (Fig. 5; Fig. S10). These wild-type recipients were of the same genetic stocks used to generate our F0 transgenics. Forty-eight hours prior to transplantation, recipients had been exposed to a sub-lethal dose of gamma-radiation to induce transient immunosuppression thereby enhancing the probability of donor cell engraftment (see Materials and Methods for details). The transient nature of this immunosuppression is demonstrated by the fact that irradiated, non-transplanted animals are viable, and their marrow flow profiles are comparable to non-transplanted animals at 60 days post-irradiation (Fig. S10). As controls, WKM cells from healthy age-matched *GFP* control animals were similarly transplanted.

For *9a+NRAS*, 12 donors were transplanted into ∼5 recipients each (producing *n*=72 recipients total). Of the 12 donors, marrow transplants from 9 induced signs of sickness in their recipients within 40 days post-transplantation (Fig. 5A). Moreover, 27% of these unwell *9a+NRAS* recipients (3/11, representing 8 donors) had marrow flow cytometry profiles with AML-like features (P gate>35%, E gate <36%), inferring the presence of LSCs in the donor marrow (Fig. 5B–E,H). Thus, the frequency of sickness in *9a+NRAS* recipients (60%) and penetrance of AML-like profiles among these (27%) is in striking agreement with the frequency of sickness and AML-like flow cytometry profiles among sick F0 *9a+NRAS* animals (∼50% and 27%, respectively). By contrast, all *NRAS* recipients (*n*=29, generated from 5 donors) and the *GFP* control recipients (*n*=20, generated from 4 donors) had normal flow profiles, remaining healthy, even after 60 days, despite evidence of engraftment in their post-mortem analysis (Fig. 5B–E,F,G). For *GFP*-cell transplanted recipients, donor cell engraftment was confirmed by detection of GFP-positive cells in control recipient flow; for *NRAS* cell recipients, engraftment was additionally confirmed by performing PCR for the *NRAS* transgene and transcript, at 60 days post transplant (Fig. S11). These data suggest that, although *NRAS* donor cells were present, their engraftment and proliferation were insufficient to establish disease or outcompete resident healthy cells. These data demonstrate that *9a+NRAS* donor marrow, but not *NRAS* donor marrow, harbour LSCs that can re-establish aggressive leukaemic disease.

## DISCUSSION

Although remission induction for *RUNX1::RUNX1T1* AMLs is generally successful, overall survival and treatment toxicity remain unacceptably poor, underscoring the need for mechanistic studies to guide new therapeutic strategies. Recent WHO classifications of myeloid neoplasia have progressively emphasised the importance of AML defining genetic abnormalities such as *RUNX1::RUNX1T1* and co-occurring molecular alterations, highlighted by removal of the requirement of over 20% blasts in marrow to define leukaemia when AML-defining genetic abnormalities are present (Khoury et al., 2022). This was based on preclinical and clinical data demonstrating that combinations of mutations drive leukaemogenesis and predict clinical behaviour and treatment response more precisely than morphological criteria alone (Duployez et al., 2016; Faber et al., 2016; Jahn et al., 2020; Papaemmanuil et al., 2016). Somatic *NRAS* mutations are frequent in *R::RT1* AML and are linked to inferior prognosis. However, *NRAS* mutations are associated with treatment failure in multiple other molecular and cytogenetic AML subtypes. Thus, *NRAS* mutations in AMLs have broad prognostic and therapeutic significance (Amatangelo et al., 2017; Christen et al., 2019; Duployez et al., 2016; McMahon et al., 2019; Winter et al., 2014). These observations prompted us to establish a F0 adult zebrafish *R::RT1* transgenic model of AML by co-expressing R::RT1 splice form *9a* and *NRAS^G12D^* in zebrafish HSPCs.

We demonstrate that expressing both *R::RT1* and *NRAS* led to a marked decrease in survival associated with marrow hypercellularity, an increase in blast/progenitor cells and a reduction in mature erythrocytes. Although this phenotype was also observed with mutant *NRAS* alone, the frequency and penetrance of this phenotype was substantially higher with co-expression of *NRAS* and *R::RT1 9a*. Moreover, transplantation of F0 *9a+NRAS* marrow cells into sub-lethally irradiated recipients induced AML with a shorter latency than its parent donor (F0 donors: 9–12 months *cf.* recipients: <40 days). Transplantability is a hallmark of AML models and is considered the gold standard to prove that the disease observed is indeed leukaemia driven by LSCs (Chopra and Bohlander, 2019). As these leukaemic features were not observed in animals expressing either *9a* or *NRAS* oncogenes alone, we hypothesise that establishment of AML stemness is mediated by the convergence of R::RT1- and NRAS-dependent activities on conserved haematopoietic regulatory networks. Future molecular analysis should reveal the identity of these targeted pathways and the mechanisms underlying their disruption.

The model's strengths include overcoming the insufficiency of *R::RT1* alone in the zebrafish system and leveraging a highly specific promoter/enhancer combination to drive spatiotemporal specificity of *R::RT1* expression (Abdallah et al., 2021; Nottingham et al., 2007; Tamplin et al., 2015). In contrast to early murine knock-in and larval zebrafish transgenic *R::RT1* models, where *R::RT1* was widely expressed outside HSPCs (Kalev-Zylinska et al., 2002; Okuda et al., 1998; Yeh et al., 2008; Yergeau et al., 1997), neither *9a* nor full-length *R::RT1* caused embryonic lethality in our system. We speculate this is because the *R1^+23^* enhancer limits expression of *R::RT1* and *9a* to HSPCs and delays expression onset until after the lethal period defined by Yeh et al. (2008), who found embryonic lethality was diminished if heat shock promotor activation occurred after 21 hpf. Consistent with this hypothesis, GFP in *R1^+23^*:GFP zebrafish only becomes apparent after this sensitive period, from 32 hpf onwards (Tamplin et al., 2015). Enhancer tissue specificity also likely explains why we did not observe *RAS*-driven oncogenesis outside the haematopoietic compartment (Le et al., 2007).

The use of a *Runx*-derived enhancer to drive oncogene expression may better recapitulate natural R::RT1/9a expression, as modelling outcomes appear to be sensitive to the oncofusion balance of endogenous RUNX1 and which HSPC subpopulations are targeted (Abdallah et al., 2021; Agrawal et al., 2020; Nafria et al., 2020; Yan et al., 2023). Using F0 animals to achieve reproducible leukaemic transformation in adult fish mimics the mosaic and competitive nature of human cancer environments and potentially reduces selection bias inherent in stable transgenic animals (Avagyan et al., 2021; Callahan et al., 2018). Furthermore, measuring altered haematopoiesis by flow cytometry allowed us to survey a much greater number of WKM cells than possible using morphology alone, improving confidence in our observations.

Our WISH analyses of transgenic larvae show that *9a* and *NRAS* have distinct effects on early haematopoiesis. In the CHT at 3 dpf, *9a* expression did not significantly increase stem cell or myeloid commitment. By contrast, *NRAS* F0 larvae showed increased *cmyb* HSPC abundance but reduced *pu.1* expression. Decreased *pu.1* could reflect impaired myeloid lineage commitment. Alternatively, NRAS-dependent *pu.1* downregulation could facilitate HSPC expansion as PU.1 also constrains HSPC proliferation to prevent stem cell expansion and exhaustion (Staber et al., 2013). The effects of *NRAS* in our model are consistent with murine and human data showing *NRAS*^G12D^ drives HSPC proliferation, thereby diminishing self-renewal potential (Li et al., 2013; Wang et al., 2011). However, HSPC proliferation was not enhanced in *9a+NRAS* larvae. Neither oncogene, singly nor in combination, altered HSPC erythroid lineage commitment in larvae. These observations suggest that the progenitor expansion and erythrocyte deficit seen in *9a+NRAS* adults are emergent properties.

AML-like disease (defined here as having >35% progenitor/precursors and transplantable disease phenotype) was preferentially associated with *9a+NRAS* coexpression. We speculate that oncogene cooperation conferred a competitive advantage within the context of the adult WKM niche, an area worthy of further investigation (Yamashita et al., 2020). While ∼27% of sick *9a+NRAS* F0s developed AML-like pathology, the remainder displayed heterogeneous cell profiles. Such phenotypic variability is potentially because we are expressing two oncogenes and that there will be animal to animal variability in the levels of 9a and/or NRAS expression. It is also possible that additional oncogenic mutations were acquired during leukaemia development, given the mutator activities of R::RT1 and oncogenic NRAS, a question to be explored in future studies.

We could not demonstrate that our phenotype correlates with a high percentage of MPO-positive immature granulocytes. This may be because our oncogene combination can only confer a subset of AML features, and additional mutations are required. For example, murine models co-expressing *NRAS^G12D^* with *R::RT1* or splice variant *9a* increased AML penetrance and reduced latency without altering the granulocytic character of the AMLs (Abdallah et al., 2021; Zuber et al., 2009). In contrast, expression of *NRAS^G12D^* alone led to monocytic myeloid leukaemias (Li et al., 2011; Wang et al., 2011; Wang et al., 2015). The early expression of *R::RT1* in our system could have contributed to our finding that MPO-positive cell frequency was not significantly enhanced in *9a+NRAS* animals, as would be expected for *R::RT1-*driven AMLs. A recent study by Abdallah et al. (2021) demonstrated the impact of *R::RT1* induction timing on AML penetrance and state of differentiation. Specifically, induction of *R::RT1* in 3-day-old mice produced AMLs without differentiation (M1), whereas later induction at 2 weeks of age favoured M2 AML (i.e. AMLs with differentiation). Induction at even later time points (4, 8 or 16 weeks) resulted in a reduced incidence of M2 AMLs and an increasing trend towards myeloproliferative disease or no disease. The choice of *R::RT1* driver may explain differences in AML penetrance and cooperating gene dependence between our model and Abdallah et al. (2021). Abdallah et al. targeted oncofusion expression to the murine HSPC population using an *R1^+24^* driver (the same *Runx1* +23 element) but fused to a basal heat shock promoter (Ng et al., 2010). Consequently, *R::RT1* expression in *R1^+24^*-positive cells was ultimately controlled by the *Rosa26* promoter after Cre^ER^-mediated recombination. In contrast, in our model, *R1^+23^* directly drives *R::RT1* expression from early embryonic life onwards. While HSPC-targeted expression of *R::RT1* alone was sufficient to drive AML in the murine model, we observed that neither *9a-* nor full-length *R::RT1* alone produced an AML phenotype in zebrafish (Fig. S3). While species-related differences may have contributed to this outcome, we speculate that our model's inherent mosaicism, combined with the relative strength of the ultimate *R::RT1/9a* expression driver (*Rosa26* promoter in the murine model, *R1^+23^* in ours), may be contributory. Single cell sequencing may offer methodologies to explore this important observation in the future.

Our finding that *9a+NRAS* impacts myeloid and erythroid adult lineages similarly to R::RT1/9a expression in other models and in humans reinforces that these oncogenes work through conserved molecular targets in zebrafish, leading to impairment of normal haematopoiesis (Kalev-Zylinska et al., 2002; Yeh et al., 2008) and offers multiple avenues for future exploration. t(8;21) AMLs with mutations in kinase signalling, chromatin modifiers or cohesins have a higher risk of relapse (Christen et al., 2019; Duployez et al., 2016). Our model provides a sensitised genetic background for co-expressing additional cohesin complex mutations such as *RAD21* to explore the underlying biology further (Jann and Tothova, 2021; Leeke et al., 2014). Different AML model systems have provided complementary insights into how oncogenic *NRAS* mutations promote AML development, revealing actionable drug targets and pathways. In an *in vitro R::RT1* cord blood model, co-expression with *NRAS^G12D^* increased replating potential and reduced apoptosis by increasing levels of anti-apoptotic factor BCL2 (Chou et al., 2011). The BCL-2 inhibitor venetoclax is being trialled in paediatric and adult AMLs. Similarly, in a murine model of inv(16) AML driven by rearrangement of CBFB (core-binding factor subunit beta), NRAS^G12D^ blocked cell death through upregulation of Bmi and the MEK pathway, the latter rendering this AML sensitive to MEK inhibitors (Xue et al., 2014). In contrast, in a *KMT2A-AF9* murine model, NRAS^G12D^ enhanced KMT2A-AF9-mediated expression of Myb, a master regulator of transcriptional programs underlying self-renewal, through upregulation of the mTOR-AKT pathway (Sachs et al., 2014). These myeloid model systems helped elucidate the diverse mechanisms underlying oncogenic cooperation and identified potential predictive biomarkers that could guide patient care. Here, we describe a novel adult zebrafish model of a common AML subtype to further unravel the molecular and therapeutic implications of oncogene cooperation.

## MATERIALS AND METHODS

### Zebrafish husbandry and ethics

All zebrafish (*Danio rerio*) strains were maintained under standard husbandry conditions and followed protocols approved by the Animal Ethics Committee of the University of Auckland (AEC22627). Wild-type (AB) zebrafish were obtained from the Zebrafish International Resource Centre (ZIRC).

### Zebrafish expression construct generation

Tol2-based expression vectors were used for transgenesis of zebrafish (Kwan et al., 2007). Middle entry vectors (pMEs) encoding *9a* and *9a+NRAS* were generated using a multi-cistronic base pME vector, pME MCS *2x P2A GFPNRAS*. This base vector was generated by inserting a customized gene block (IDT) between the *att* sites of pENTR/D-TOPO (Invitrogen) (Fig. S1). This insert consists of the two copies of the *P2A* (*porcine teschovirus*-*1 2A*) ‘ribosome-skipping’ sequence, each proceeded by unique restriction enzyme sites for inserting GoIs. The *P2A* sequences are followed by an in-frame ORF encoding *EGFP* fused to human *NRAS^G12D^ (GFPNRAS^G12D^*, abbreviated here to *NRAS*) and a termination codon. To generate pME *9a+NRAS (9a-P2A-GFPNRAS^G12D^),* a Smi1-Zra1 PCR fragment encoding *9a* with a canonical Kozak sequence (Grzegorski et al., 2014) but no termination codon was generated using *R::RT1 9a* gene-specific primers (Table S1) and pMIG AE9a plasmid as the *R::RT1* DNA template (Desai et al., 2020). The *9a* PCR fragment was then cloned into the Smi1 and Zra1 site of pME *2 x P2A NRAS* (Fig. S1). pME *9a (9a-P2A-GFP)* was derived from pME *9a+NRAS* by cutting with Xho1 and Pac1 to excise *NRAS*, followed by end-filling and re-ligation. To generate pME *NRAS,* a PCR fragment containing *GFPNRAS^G12D^* with a canonical Kozak and a stop codon, was generated using gene-specific primers (Table S1) and pME *2x P2A NRAS* as the template. This fragment was then cloned into pENTR/D-TOPO (Thermo Fisher Scientific), as per the manufacturer's instructions. The final pDEST expression constructs were generated by performing Gateway LR reactions (Life Technologies), as per the manufacturer's instructions, using 5′ entry vector p5E *R1^+23^* (a gift from Owen Tamplin and Leonard Zon, Addgene plasmid # 69602; http://n2t.net/addgene:69602; RRID:Addgene_69602), 3′ entry vector p3E *polyA* (zebrafish Tol2kit plasmid ID 191), destination vector pDEST Tol2 CG2 (ID 204; with transgenesis heart marker *cmcl:EGFP*) and one of the following middle entry vectors: pME *GFP* (ID 180), pME *9a+NRAS*, pME *NRAS*, pME *9a* or pME *R::RT1-9a-NRAS*. The presence of a *P2A* sequence between *9a* and *NRAS* ORFs enabled the generation of discrete 9a and GFPNRAS translational products (Liu et al., 2017; Szymczak et al., 2004). In *9a+NRAS* vectors*, NRAS* was strategically placed downstream of *9a* and the *P2A* sequence, exploiting the second position effect observed with bi-cistronic constructs, whereby the gene in the second position is translated at a lower frequency than that in the first, thus serving to reduce NRAS expression levels and the likelihood of NRAS dominating any resultant phenotype (Liu et al., 2017). *IRES-GFP*-based expression constructs were generated by recombining p5E *R1^+23^*, pME full-length *R::RT1* or *9a*, p3E-*IRES-EGFPpA* (ID 195) and pDEST Tol2 CG2 (ID 204).

### Generation of F0 mosaic transgenics

Transgenic zebrafish were generated by micro-injection of pDEST expression vectors DNA (Fig. 1) into one-cell stage wild-type AB embryos (Suster et al., 2009). Wild-type embryos for injection were collected from naturally spawned wild-type zebrafish. Injections were performed using pre-pulled microinjection needles calibrated to inject at 1 nl/pulse. The injection cocktail consisted of pDEST expression plasmid DNA (final concentration of 25 ng/µl), transposase mRNA (25 ng/µl) and Phenol Red as a visible marker. At 24 h post injection, embryos were screened for GFP expression in the heart. Positive embryos were either used for WISH experiments or raised to adulthood using standard zebrafish husbandry procedures.

### WISH

WISH was performed on 4% PFA-fixed embryos as previously described (Thisse and Thisse, 2008). RNA probes were generated by linearisation of vectors containing the relevant cDNA sequence (see Table S2 for probe sequence information). Digoxigenin-labelled antisense probes were synthesised using an RNA Labelling Kit (SP6/T7; Roche). Staining was revealed with NBT/BCIP or INT/BCIP substrate (Roche). Two technical replicates were performed for each probe (∼30 animals/genotype/probe/replicate). WISH embryos were imaged in 100% glycerol, using a Zeiss Axio Zoom microscope. CHT staining intensity was quantified as described previously (Dobrzycki et al., 2020) and scored blind of genotype. Specifically, all technical replicates were imaged under identical lighting and exposure conditions. For each animal a ‘corrected CHT signal’ was obtained (the raw CHT signal ROI − a background pixel intensity ROI of the same size). For each probe, the mean ‘corrected CHT signal’ for GFP was calculated and used to normalise the CHT values for all other genotypes of that technical replicate. These normalised ‘Signal Intensity’ values were plotted in Fig. 1 and Fig. S2.

### Zebrafish whole kidney marrow cell collection

Sick or well zebrafish adults were humanely euthanised by placing them in an ice bath. To isolated whole kidney marrow (WKM) cells, kidney marrows were dissected out then transferred to cold FACS buffer consisting of 0.9 x PBS (Gibco) and 5% FBS (Moregate Biotech). Marrow cells were manually dissociated by pipetting up and down then passing through a 40 µM cell strainer (Falcon).

### Cell flow cytometry analysis and sorting

Immediately prior to flow cytometry, 1 µl 1% Propidium Iodide (Sigma-Aldrich) was added to dissociated WKM cells in FACS buffer and cells were briefly vortexed to mix. Cells (100,000 events) were analysed for size (Forward Scatter, FSC) and granularity (Side Scatter, SSC) using a BD LSRII Flow Cytometer (Becton Dickinson Biosciences). Gates were drawn around the distinct populations of erythrocytes, myelomonocytes, lymphocytes and precursors/progenitors (Traver et al., 2003). After flow analysis, the remaining cells were either used for smears (applied to slide manually or using a cytospin machine), RNA isolation and/or cell transplantation. For cell sorting, gated populations were sorted into either 25 µl FACS buffer 1 mM EDTA (myelomonocytes, progenitor/precursors, lymphocytes, R1^+23^:GFP-positive cells) or FACS buffer with 2% (v/v) heparin (erythrocytes). All flow cytometry data was acquired using BD FACSDiva software (v6.1.1) and analysed with FlowJo (10.8.1).

### Cytospin

Sorted cells (1×10^5^) in FACS Buffer [1 mM EDTA or 2% (v/v) heparin] or unsorted WKM cells (3×10^5^) in FACS buffer, were spun onto slides at 120×g, 4°C using the Aerospray^®^ Hematology Pro (ELITech Group Inc.) and allowed to dry overnight before staining with May Grünwald-Giemsa stain (ELITech Group Inc.) applied using the cytospin machine.

### Myeloperoxidase staining

WKM smears were air dried for 24 h before staining for myeloperoxidase. 3,3′-Diaminobenzidine (DAB, Sigma-Aldrich) was added to MPX Buffer (50 mM Tris-HCl pH 7.4) and mixed well prior to adding 3% H_2_O_2_. Slides were then flooded with cold buffered formalin acetone (5 mM Na_2_HPO_4_, 30 mM KH_2_PO_4_, 45% (v/v) acetone, 25% (v/v) 37% formalin) for 30 s before rinsing in distilled water. Slides were immersed in the DAB solution for 15 min at room temperature, then rinsed with distilled water and counterstained in Mayer's modified Haematoxylin Solution (Abcam). Following imaging, cells were counted using ImageJ (Fiji).

### Histology

Adult zebrafish were euthanised on ice before placing in 4% (v/v) PFA (EMS) in the fridge from 4 days to 2 weeks. After this, all PFA was removed and replaced with 0.25% (w/v) EDTA to decalcify for 5–7 days. Post decalcification, animals were left in 75% EtOH for 3–7 days before being embedded and sectioned (10 µM thickness). Sagittal sections were then stained in Haematoxylin and Eosin.

### Imaging of live adults and tissue sections

To image live adult zebrafish, animals were anaesthetised in 4% (v/v) Tricaine (Sigma-Aldrich). Imaging was performed using a Leica MZ10F Microscope. Images were processed using ImageJ (Fiji). Sections and sorted or unsorted kidney marrow slides were imaged using the Zeiss Axio Imager M2 (ZEISS) and MetaMorph^®^ software (Molecular Devices, LLC.).

### RT-PCR

WKM cells were isolated and resuspended in FACS buffer as described for flow cytometry. A minimum of 500,000 cells were recovered by centrifugation and the pellet resuspended in Qiagen RLT buffer supplemented with 2-mercaptoethanol (Merck). RNA was isolated using Qiagen Rneasy Mini columns according to the manufacturer's instructions. The purified RNA was then Dnase-treated using ezDNase Enzyme (Invitrogen). A portion of Dnase-treated RNA was kept aside as a “No RT” control, while the remainder was used in first strand cDNA synthesis reactions with Superscript IV (Invitrogen), priming with random hexamers oligos (Invitrogen). PCR was performed using the KAPA2G Robust HotStart PCR Kit (Kapabiosystems) with 2 µl of cDNA synthesis reaction (+RT) or No RT template, per PCR reaction and the gene-specific primers for *9a*, *NRAS* (this study) or *ef1a* (Oehlers et al., 2011) (Table S3). PCR products were run on Tris Acetate EDTA pH 8-buffered 10% polyacrylamide gels alongside a GeneRuler 50 bp DNA ladder (Thermo Fisher Scientific).

### Transplantations

Healthy wild-type zebrafish were gamma irradiated with a sublethal dose of 18 Gy, 48 h prior to transplantation. Whole kidney marrows were isolated from prospective donor animals. WKM cell resuspension counts were determined by counting Trypan Blue-stained cells manually on a haemocytometer. Irradiated recipients (5–10 animals) were individually anaesthetised in 4% (v/v) Tricaine then injected intra-peritoneally with 3×10^5^ of WKM cells per recipient.

### Statistics

The data analysed corresponds to animals from 2 to 4 injected batches per genotype, generated over the course of 4 years. All statistical analysis was done using GraphPad Prism version 9 for the Mac (GraphPad Software). Statistical tests used in analysis were unpaired *t*-test, one-way analysis of variance (ANOVA) with Tukey's multiple comparison, Kruskal–Wallis or a Log-rank (Mantel-Cox) test.

## Supplementary Material

10.1242/biolopen.060523_sup1 Supplementary information
