# Supplementary material for: Mutational cooperativity of RUNX1::RUNX1T1 isoform 9a and oncogenic NRAS in zebrafish myeloid leukaemia
Source: Biol Open. 2024 Aug 30;13(9):bio060523. doi: 10.1242/bio.060523 (PMC11381922; doi:10.1242/bio.060523)
Supplement: Supplementary information [file biolopen-13-060523-s1.pdf]

A

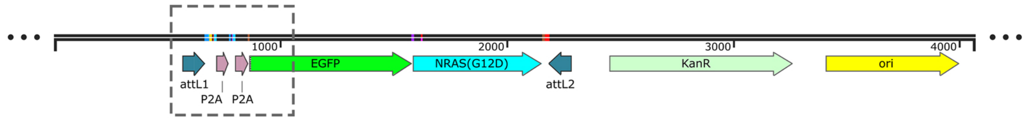

B

..atgccaactttgtacaaaaaagcaggctccgcggccgccccttcacc**atttaaat**tgctcg
GTCGACGGCAGCGGCGCCACAAACTTCTCTCTGCTAAAGCAAGCAGGTGATGTTGAAGAAAACC
CCGGGCCT**CATATG**agccta**GACGTC**GGCAGCGGCGCCACAAACTTCTCTCTGCTAAAGCAAGC
AGGTGATGTTGAAGAAAACCCCGGGCCTTCCGGAATGGTGAGCAAGGGCG…

Fig. S1. Organisation and sequence of the basic, multi-cistronic, gateway middle entry vector, pME MSC 2 x P2A GFPNRAS, used in the generation of transgene expression constructs.
(A) Schematic showing the general organisation of the cloning region. *attL1* and *attL2* correspond to the pME vector backbone *att* recombination sites. Encoded protein sequences are *Porcine teschovirus-1* 2A (P2A); enhanced GFP (EGFP); human NRAS (NRAS, NM_002524.5) modified to encode the G12D (gGt to gAt) mutation. (B) Corresponding nucleotide sequence for the region boxed in A. Protein encoding sequence (uppercase); non-coding sequence (lowercase), *attL1* (blue text). The unique restriction sites (bold text) enable directional cloning of up to two GoIs, with an in-frame *P2A* coding sequence following each (pink highlighting). Each P2A peptide is proceeded by Gly-Ser-Gly (grey highlighting) for enhanced ribosome tracking. EGFP is indicated by green highlighting. The *NRAS* ORF downstream of *EGFP* (not shown) is flanked by unique restriction sites (Xho1, Pac1) so can be excised.

Biology Open • Supplementary information

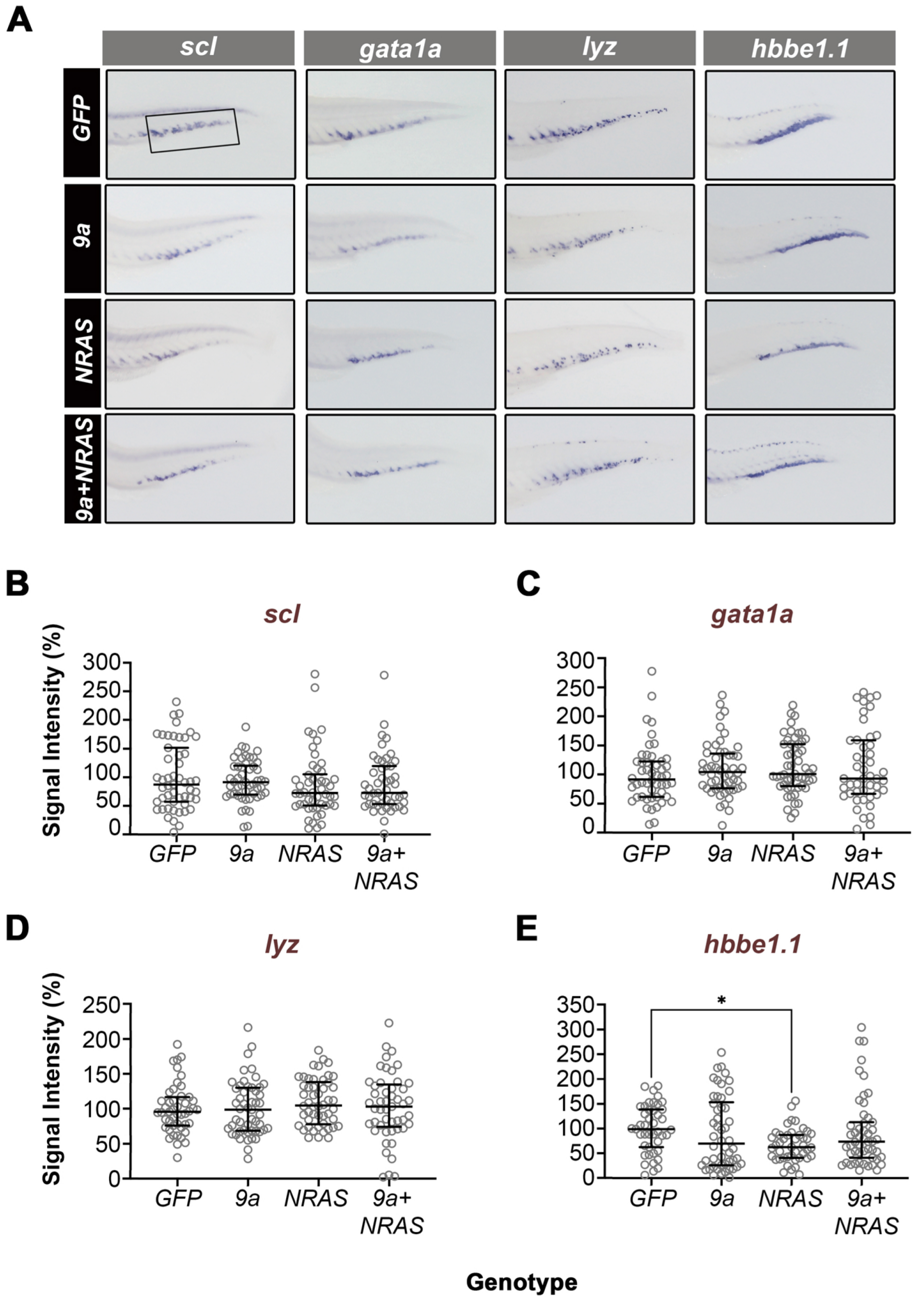

Fig. S2. Impact of oncogene expression on HSPC regulators and haematopoietic terminal markers.

(A) Images of WISH F0 transgenic animals at 72 hpf, excluding data presented in Fig.1. Probe used (top) and genotype (left) are indicated (see Table S2 for probe details). (B-E) Quantitative analysis of *in situ* probe signal intensity in the CHT (boxed region; see Materials and Methods for details of the quantification process). Each circle corresponds to the normalized staining pixel intensity of a single animal for the probe indicated. The total number of animals shown per probe and genotype (~50-60 animals) corresponds to two technical replicates. Statistical test: Kruskal-Wallis (if non-Gaussian distribution) or ANOVA (if Gaussian distribution).

Biology Open • Supplementary information

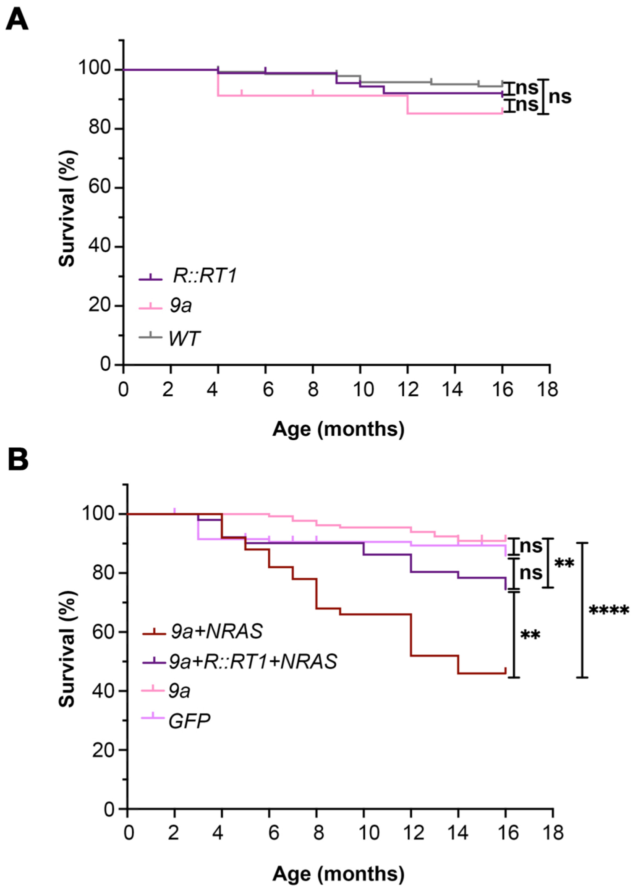

Fig. S3. Kaplan-Meier survival plots for F0 transgenics expressing full length *R::RT1*, alone or with *9a* and *NRAS*.

Survival plots of (A) transgenic F0s expressing *R1*$^{+23}$-driven full length- or *9a R::RT1* isoforms. Number of animals per genotype, n: *Wild type (WT)*, 144; *R::RT1*, 92; *9a*, 23. Statistical test: Log-rank (Mantel-Cox) test: ns, not significant. (B) F0s co-expressing *9a +R::RT1+NRAS*, compared to F0s expressing *9a* alone or *9a+NRAS*. The rationale for *R::RT1* and *9a* co-expression is based on the observation that patients express both forms. Number of animals, n: *9a+R::RT1+NRAS*, 51; other genotypes: as per A. Statistical test: Log-rank (Mantel-Cox) test: **, p = 0.0037 (9a+R::RT1+NRAS vs. 9a), p = 0.0032 (*9a+NRAS* vs. *9a+R::RT1+NRAS*); ****, p < 0.0001; ns, not significant.

Biology Open • Supplementary information

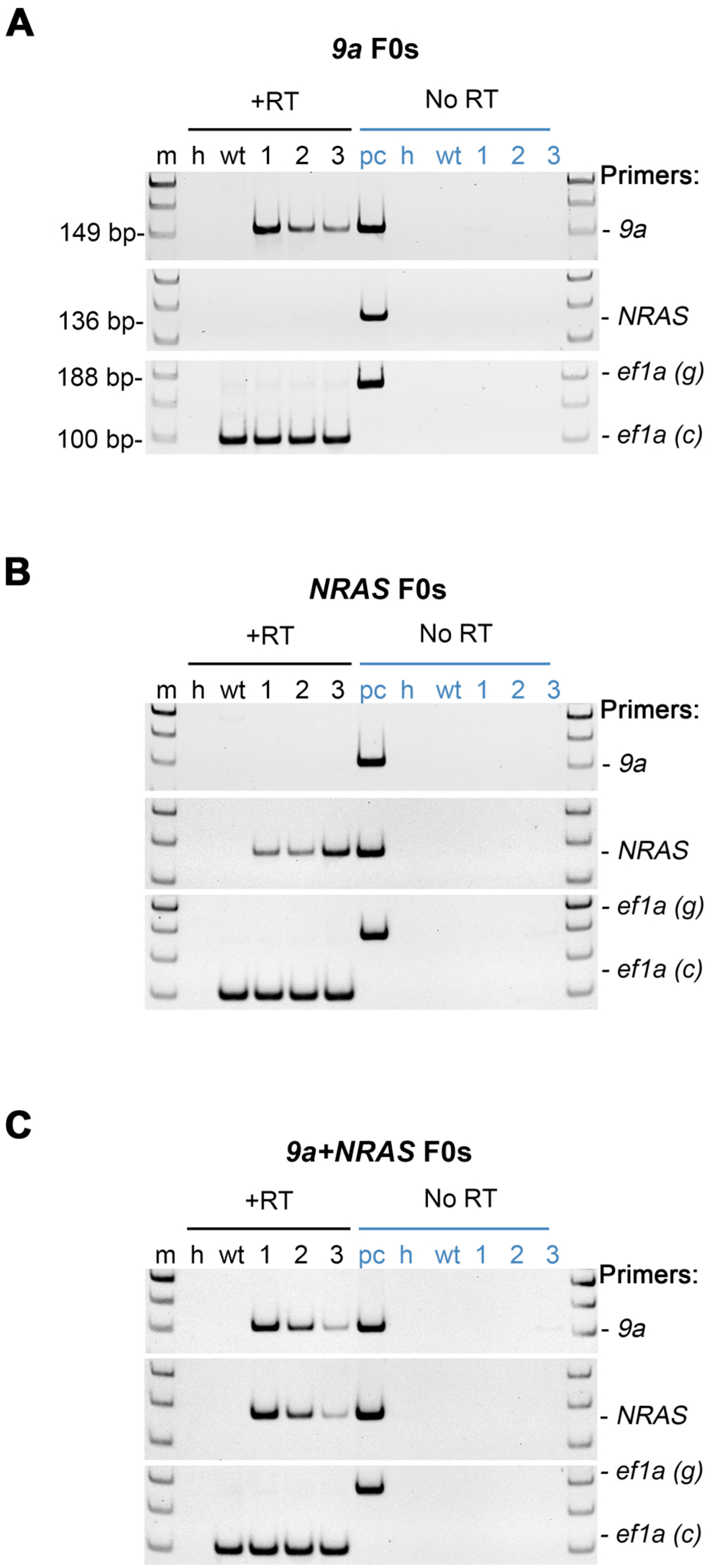

Biology Open • Supplementary information

Fig. S4. RT-PCR confirmation of transgenes expression in WKM cells from F0 animals.

(A-C) Polyacrylamide gels of PCR products generated with gene-specific primers (relevant gene primers indicated to right of gel) and templates from the genotypes indicated (lanes). RT, reverse transcribed template (cDNA); No RT, RNA template that is Dnase-treated but not reverse transcribed; h, water; wt, wild type; pc, PCR positive control for the primers used (with a template of genomic DNA from the relevant transgenic genotype). Lanes marked 1, 2 and 3, are representative F0 animals of the relevant genotype; m, molecular marker (GeneRuler 50 bp ladder, Invitrogen). Expected PCR product sizes: *9a* (149 bps), *NRAS* (136 bps), internal control for PCR: *ef1a* (g), genomic product (188 bps), *ef1a* (c) cDNA product (101 bps). See Table S3 for primer sequences.

Biology Open • Supplementary information

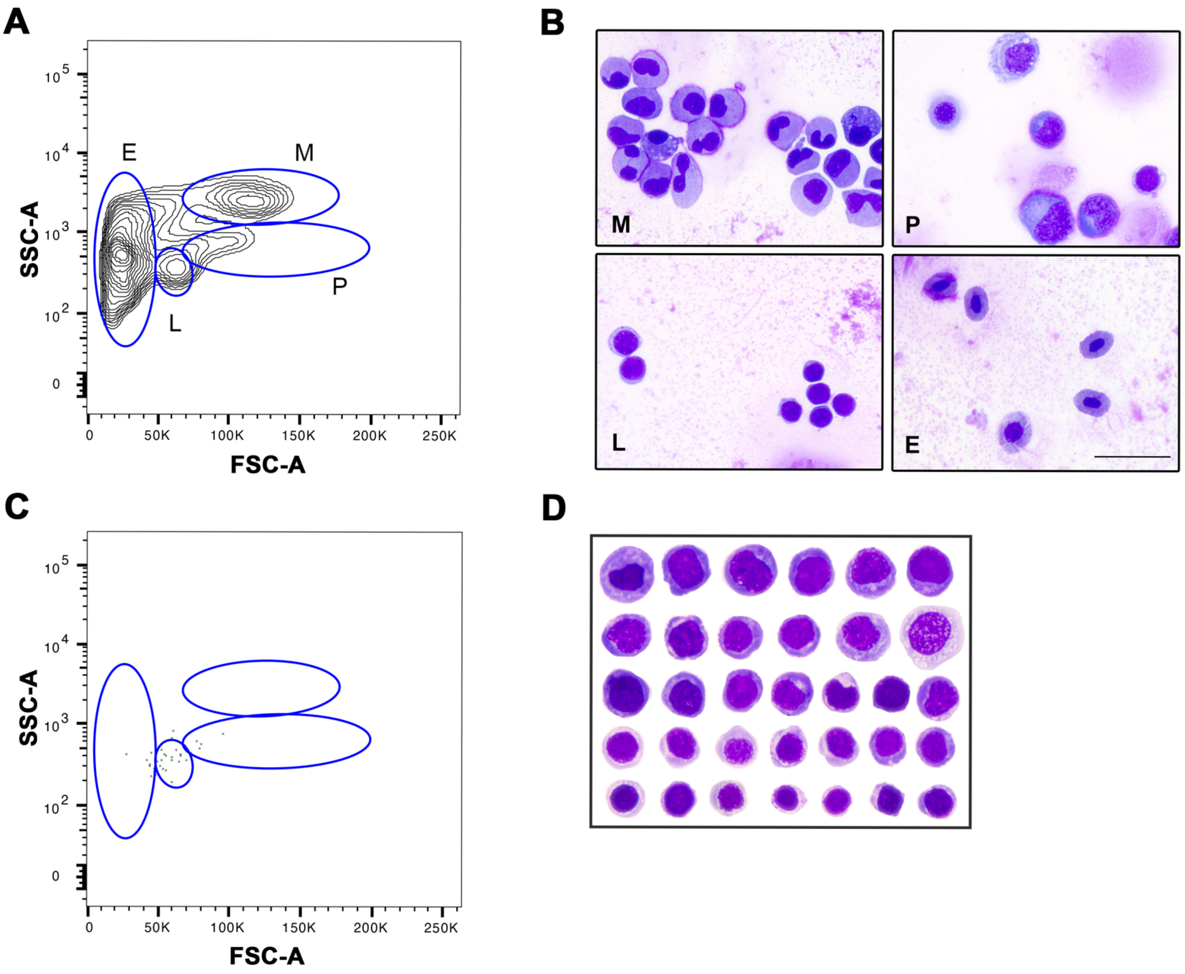

Fig. S5. Morphology of FSC/SSC-gated WKM populations and of WKM *R1+23:GFP*-positive cells.

(A) Forward Scatter (FSC-A) versus Side Scatter (SSC-A) plot of a WKM cell suspensions from an adult *R1+23:GFP* F0 zebrafish. The gates drawn conform to the natural grouping of cells in the scatter plot and correspond to the following cell groups: erythrocytes, E; myelomonocytes, M; precursors/progenitors, P; and lymphocytes, L. (B) May-Grünwald Giemsa staining of cells sorted from the gates shown in A. (C) Distribution of WKM GFP-positive cells, relative to the gates in A.(D) Montage of May-Grünwald Giemsa-stained GFP-positive cells sorted from adult *R1+23:GFP* F0 WKM cell suspensions. Magnification = 630x; Scale bar = 20µM.

Biology Open • Supplementary information

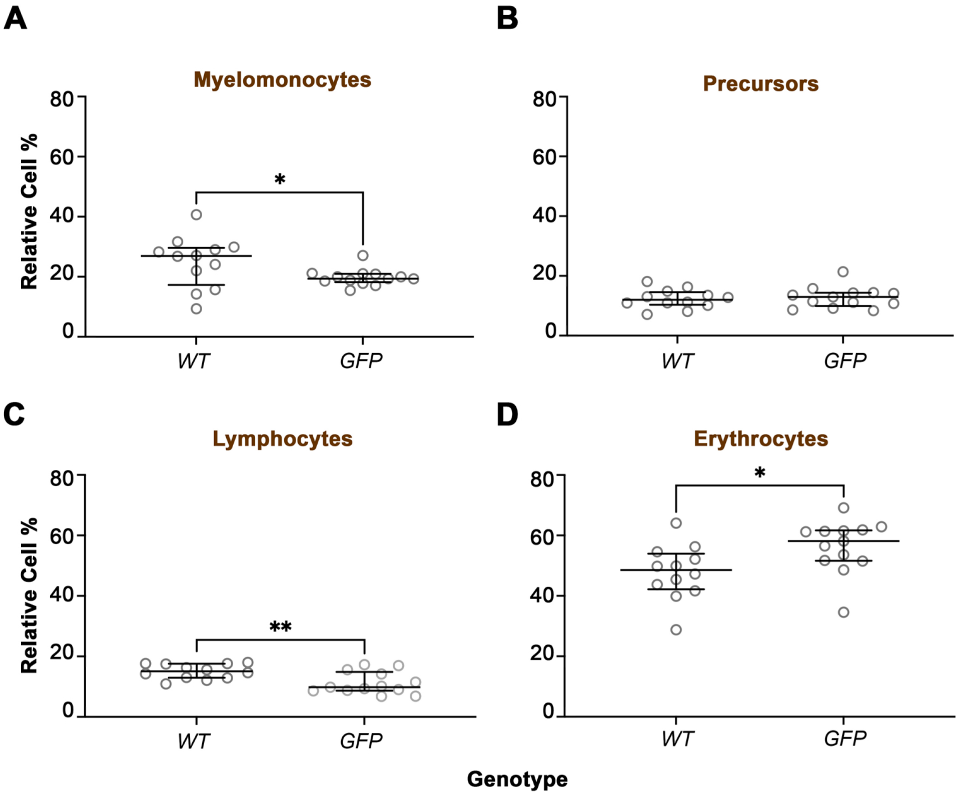

Fig. S6. Comparison of *WT* and F0 *R1*$^{+23}$*:GFP* WKM flow cytometry. Quantitative analysis of WKM cells types from the wild-type (*WT*) background used to generate the transgenic F0 genotypes, including *R1*$^{+23}$*:GFP* F0s. FSC/ SSC analysis was performed on age-matched animals. (A-D) show the relative percentages of cells in the four gated populations for each genotype. For each animal, the cell percentages were normalised so that the sum of all gates came to 100%. Each data point shown on the graph corresponds to a single animal. Number of animals per genotype, n: *WT*, 12; *GFP*, 13. Statistical analysis: unpaired t-test with the Q1, Q2 (median) and Q3 shown. Graphs show only those comparisons that were statistically significant. *, p = 0.0450; **, p=0.0049.

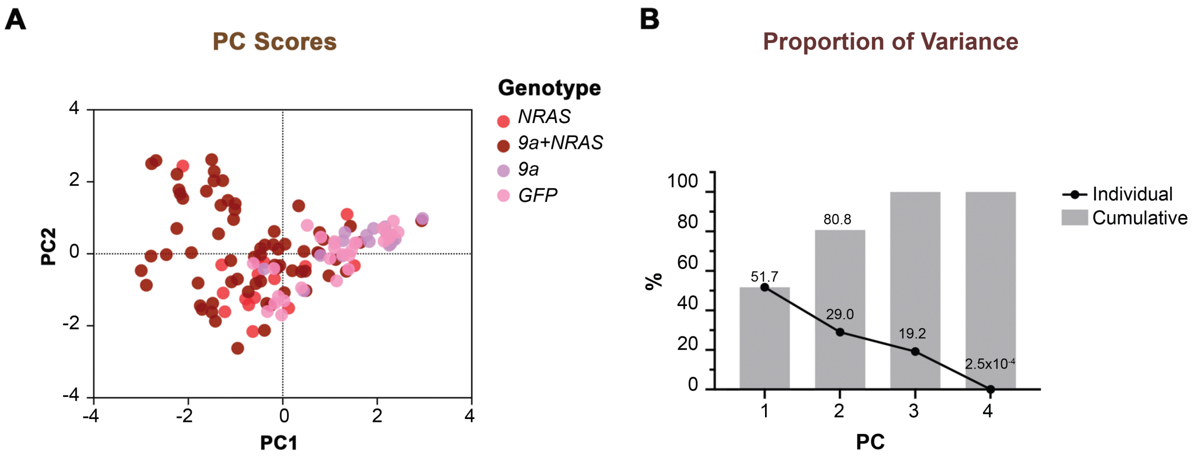

Fig. S7. Principle component analyses of F0 flow cytometry data.
(A) PCA of flow data based on all four gated cell populations show in Fig. 3.(B) PCA histogram showing the contribution of PC1-4 to the total variance, with PC1 and PC2 cumulatively accounting for 80.76% of the variance.

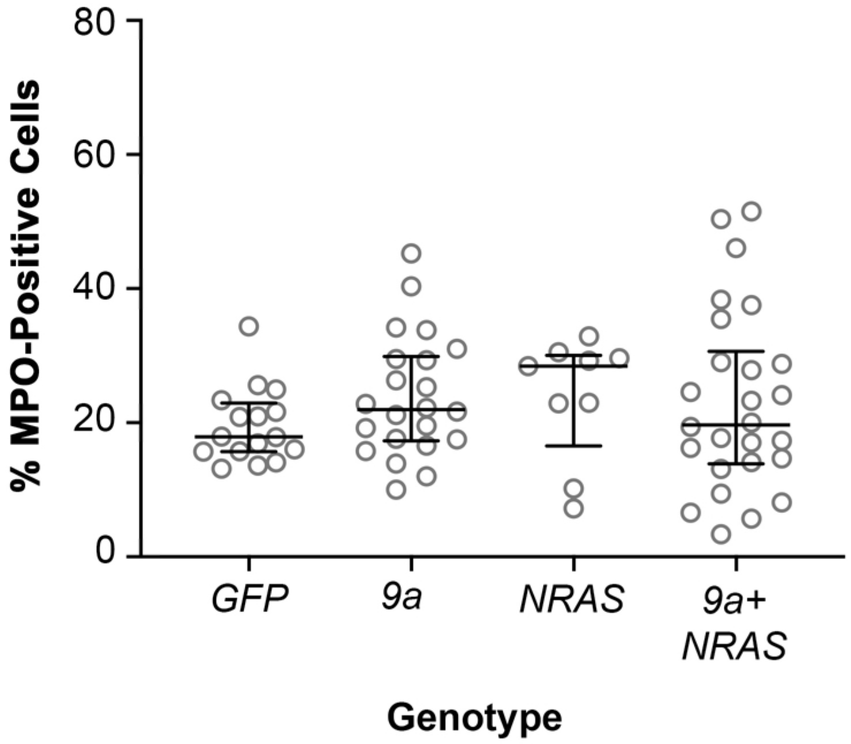

Fig. S8. Percentage of MPO-positive cells in WKM of F0 genotypes. WKM cells were isolated from age-matched F0 animals of the genotype shown, smeared on to slides then stained *in situ* for myeloperoxidase enzyme (MPO) activity (see Materials and Methods). Cells were counted and the percentage of MPO-positive cells calculated. Each data point corresponds to the MPO percentage for a single animal. Number of animals per genotype, n: *GFP*, 16; *9a*, 22; *NRAS*, 9; *9a+NRAS*, 26. Statistical analysis: Kruskal-Wallis. Medians (Q2) and interquartiles (Q1 and Q3) are shown. There was no significant difference between genotypes.

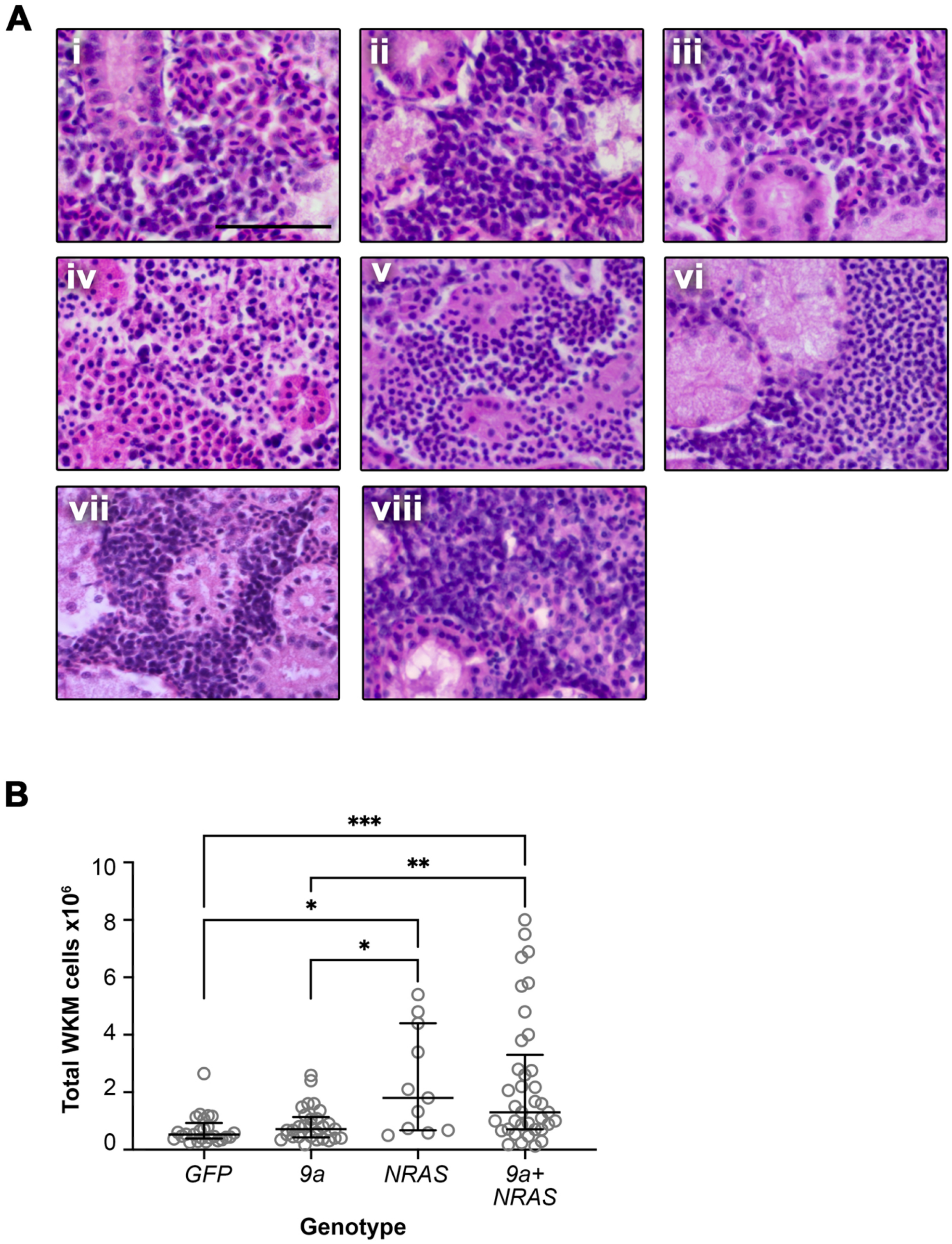

Fig. S9. WKM cellularity in F0 transgenics.

(A) H&E-stained sections from eight sick *9a+NRAS* F0 animals (not presented in Fig. 4), illustrating the spectrum of cell densities observed, with only viii being hypercellular. Magnification = 630x; Scale bar = 20μM. (B) WKM cell counts obtained by counting cells from freshly isolated WKM preparations, from the genotype indicated. Each circle corresponds to a single animal. Statistical Test: One-Way ANOVA: *, p=0.0216, 0.045; **, p=0.0014 ; ***, p= 0.0005

Biology Open • Supplementary information

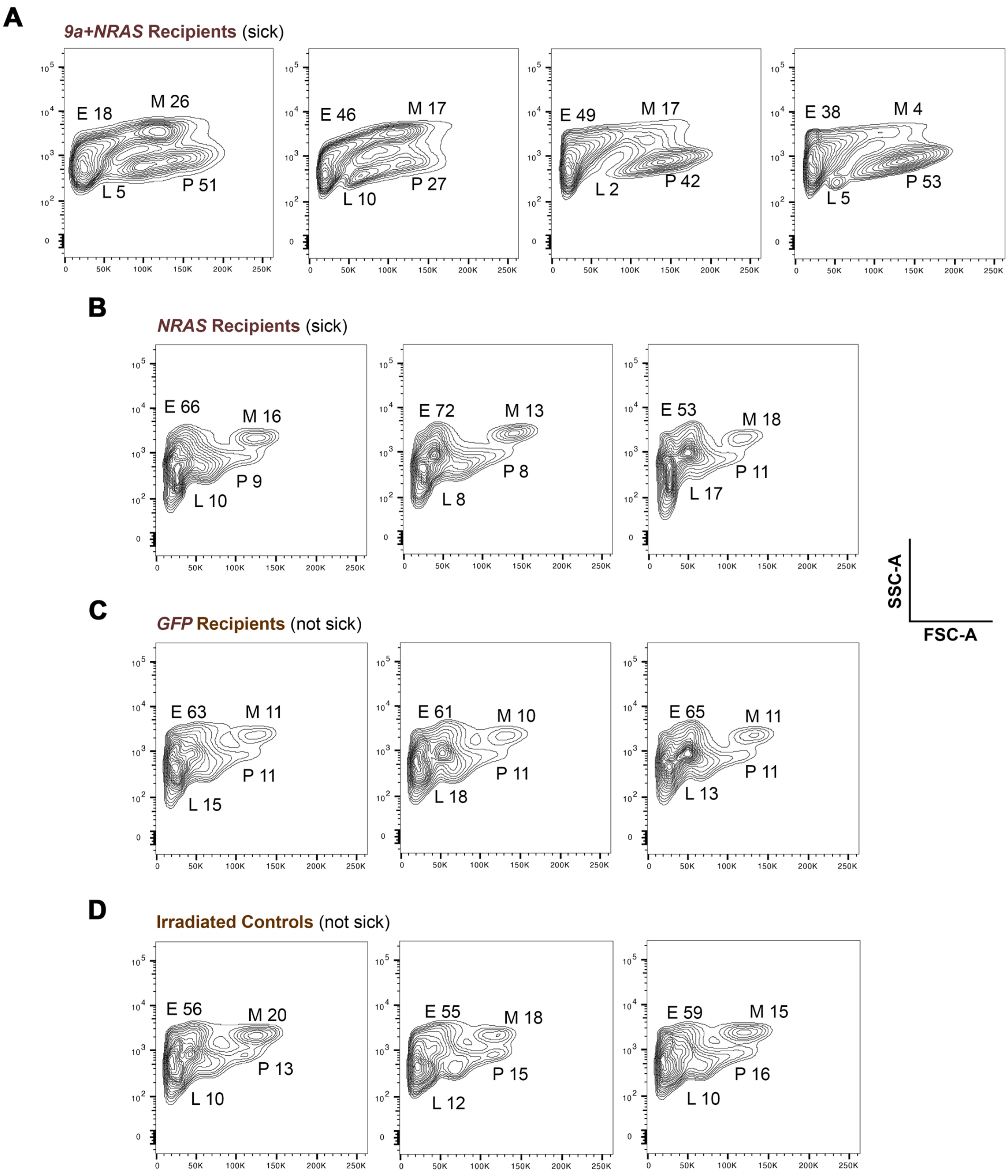

Fig. S10. WKM flow cytometry profiles of 1° recipients.

FSC/SSC flow cytometry profiles of individual animals transplanted with cells of the genotype indicated or non-transplanted controls (irradiated but not transplanted animals), excluding those presented in Fig. 5. The M, P, L and E numbers correspond to the percentage of cells in these respective gates. For *9a+NRAS* transplant recipients, onset of sickness and subsequent analysis ranged from 19 to 30 days post-transplantation. All non-sick animals shown were euthanised for analysis at 60 days post-transplantation.

Biology Open • Supplementary information

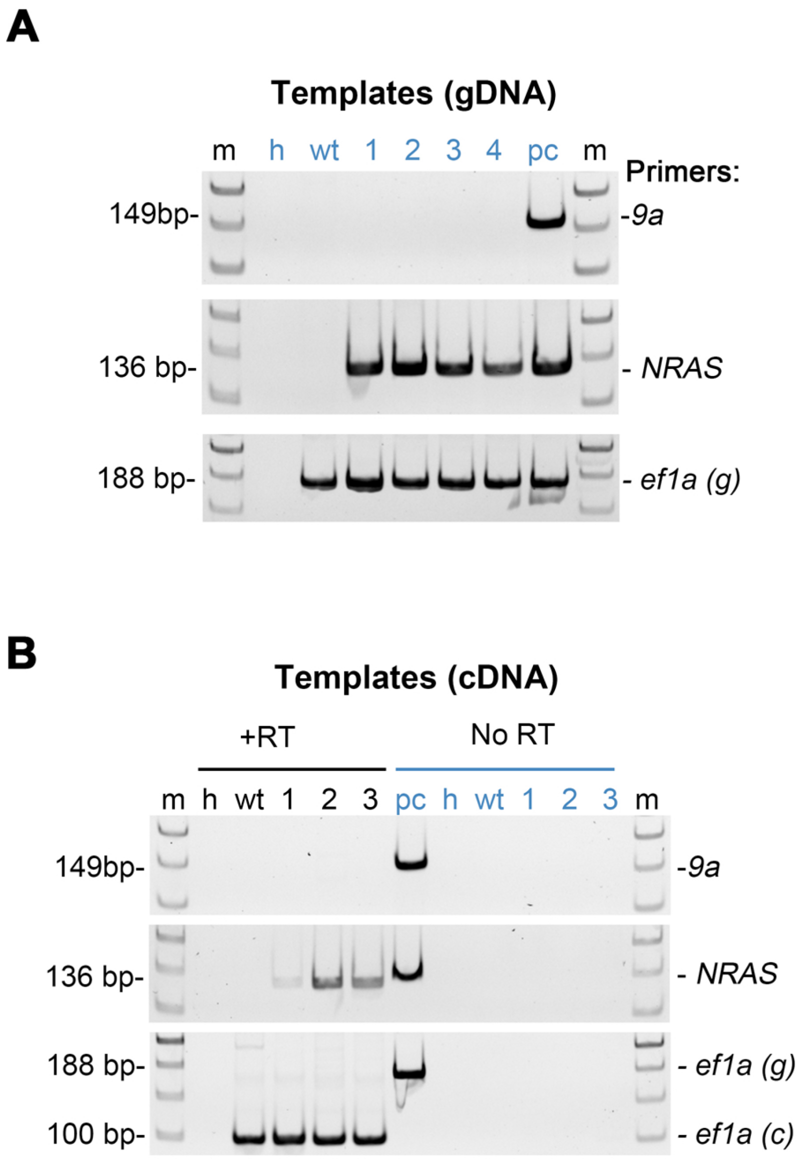

Fig. S11. *NRAS* transplant recipients harbour *NRAS*-expressing cells but do not develop leukaemia. Polyacrylamide electrophoresis of PCR products generated using gene-specific primers (for *9a, NRAS* or *ef1a*) from the genotype templates indicated. (A) PCR with genomic DNA templates. (B) PCR with RT/No RT templates. In A, lanes numbered 1 to 4 correspond to genomic DNA from individual animals; in B, lanes correspond to their respective RT/No RT templates. See Fig. S4 legend for labelling key and Table S3 for primer sequences.

Biology Open • Supplementary information

Table S1. Primers used for constructing pME oncogene vectors. Relates to Fig. 1A – zebrafish expression constructs.

Primer name	Primer sequence	Application
Smi Kz *R::RT1/9a* ATG F	TGTGTATTTAAATCACCATGCGTATCCCCG	Generation of a PCR fragment encoding the *9a* ORF. This fragment was inserted into pME 2x P2A *NRAS* to generate pME *9a+NRAS*
Zra *R::RT1/9a* NO STOP R	TATATGACGTCTCCTAGTGCAACTGGGTCT	
Inner Kz *GFP* F	CACCATGGTGAGCAAGGGC	Generation a PCR fragment encoding GFPNRAS^{G12D} with a Kozak sequence. This was cloned into pENTR-D TOPO (Life Technologies) to generate pME *NRAS*.
NRAS STOP Pac R	TGATCTTAATTAATTACATCACCACACATGGCAAT	

Table S2. WISH Probes Relates to Fig. 1 and Fig. S2

Gene	Ref sequence	Probe size and gene region
cmyb	NM_001309822.1	Last 366 ntds of ORF
pu.1	NM_198062.2	Full transcript (1062 ntds)
cepba	BC056548.1	Last 486 ntds of ORF
scl	AF045432.2	Full transcript (2630 ntds)
gata1a	NM_131234.2	Full transcript (1580 ntds)
lyzC	NM_139180.1	Full transcript (596 ntds)
hbbe1.1	NM_198073.2	Full transcript (583 ntds)

Table S3. RT-PCR primers
Relates to Fig. S4 (F0s) and Fig. S10 (*NRAS* transplant recipients)

Primer name	Sequence	Expected product size (bps)
R::RT1 9a 437 F	CTGACCATCACTGTCTTCACAAACC	149
R::RT1 9a 567 R	GATTGCGTCTTCACATCCACAGG	
NRAS 363 F	ACAAGGACAGTTGATACAAAACAAGC	136
NRAS 476 R	GGTACTGGCGTATTTCTCTTACC	
zf ef1a 638 F	TGCCTTCGTCCCAATTTCAG	188 (genomic) or 101 (cDNA)
zf ef1a 719 R	TACCCTCCTTGCGCTCAATC	

R::RT1, human *RUNX1::RUNXT1*; *NRAS* refers to human *NRAS* (NM_002524.5); zf *ef1a* zebrafish (accession number NM_131603.3).
